# Contribution of *FKBP5* Genetic Variation to Gemcitabine Treatment and Survival in Pancreatic Adenocarcinoma

**DOI:** 10.1371/journal.pone.0070216

**Published:** 2013-08-01

**Authors:** Katarzyna A. Ellsworth, Bruce W. Eckloff, Liang Li, Irene Moon, Brooke L. Fridley, Gregory D. Jenkins, Erin Carlson, Abra Brisbin, Ryan Abo, William Bamlet, Gloria Petersen, Eric D. Wieben, Liewei Wang

**Affiliations:** 1 Molecular Pharmacology and Experimental Therapeutics, Mayo Clinic, Rochester, Minnesota, United States of America; 2 Biochemistry and Molecular Biology, Mayo Clinic, Rochester, Minnesota, United States of America; 3 Division of Biomedical Statistics and Informatics, Mayo Clinic, Rochester, Minnesota, United States of America; University of Nebraska Medical Center, United States of America

## Abstract

**Purpose:**

FKBP51, (*FKBP5*), is a negative regulator of Akt. Variability in *FKBP5* expression level is a major factor contributing to variation in response to chemotherapeutic agents including gemcitabine, a first line treatment for pancreatic cancer. Genetic variation in *FKBP5* could influence its function and, ultimately, treatment response of pancreatic cancer.

**Experimental Design:**

We set out to comprehensively study the role of genetic variation in *FKBP5* identified by Next Generation DNA resequencing on response to gemcitabine treatment of pancreatic cancer by utilizing both tumor and germline DNA samples from 43 pancreatic cancer patients, including 19 paired normal-tumor samples. Next, genotype-phenotype association studies were performed with overall survival as well as with *FKBP5* gene expression in tumor using the same samples in which resequencing had been performed, followed by functional genomics studies.

**Results:**

In-depth resequencing identified 404 *FKBP5* single nucleotide polymorphisms (SNPs) in normal and tumor DNA. SNPs with the strongest associations with survival or *FKBP5* expression were subjected to functional genomic study. Electromobility shift assay showed that the rs73748206 “A(T)” SNP altered DNA-protein binding patterns, consistent with significantly increased reporter gene activity, possibly through its increased binding to Glucocorticoid Receptor (GR). The effect of rs73748206 was confirmed on the basis of its association with *FKBP5* expression by affecting the binding to GR in lymphoblastoid cell lines derived from the same patients for whom DNA was used for resequencing.

**Conclusion:**

This comprehensive *FKBP5* resequencing study provides insights into the role of genetic variation in variation of gemcitabine response.

## Introduction

Pancreatic adenocarcinoma is one of the most deadly of cancers, with a five year survival after diagnosis of about 5% [1], [2]. That is mainly due to the fact that in the early stages of pancreatic cancer development it often does not cause symptoms, and later the symptoms are nonspecific and varied [3]. Therefore, pancreatic cancer is not diagnosed until it is advanced. Currently, the first line treatment of choice for patients with pancreatic cancer is gemcitabine [4]. However, response rates to gemcitabine vary widely [5]. Previous pharmacogenetic studies have focused on genes in the gemcitabine metabolism pathways and demonstrated that either expression or single nucleotide polymorphisms (SNPs) present in those genes could only explain a portion of the observed variability in drug response [5], [6], [7]. Recently, using a genome-wide approach with 197 human lymphoblastoid cell lines (LCLs) model system, we identified *FKBP5* as a top candidate that was significantly associated with sensitivity to this antineoplastic agent [7]. Variation in *FKBP5* expression alone accounted for 14% of the variation in gemcitabine IC_50_ values observed in these LCLs while all 17 of the genes in gemcitabine metabolism pathway combined accounted for only 27 percent of the variation [7].

FKBP51 belongs to a family of large immunophilins, and it catalyzes the conversion of the cis and trans isomers of peptide bonds with the amino acid proline, a reaction that is important for protein folding [8]. FKBP51 is encoded by the gene, *FKBP5* [9]. Our previous studies suggested that the level of *FKBP5* expression is associated with variation in chemosensitivity to gemcitabine as well as other antineoplastic agents [7]. Subsequent studies revealed that FKBP51 functions as a scaffolding protein promoting the interaction between Akt and PHLPP [10]. Specifically, FKBP51 acts as a negative regulator of the Akt pathway and, under the genotoxic stress, directs cells towards apoptosis [10]. We also showed that *FKBP5* expression level could potentially be used as a biomarker for treatment selection of gemcitabine with or without Akt inhibitors using pancreatic cancer xenograft mice [11].

In the current study, we hypothesized that genetic variation in *FKBP5* might contribute to regulation of its expression, thus contributing to gemcitabine response and ultimately affecting patient survival. Therefore, we set out to identify genetic variation in *FKBP5* by performing Next Generation resequencing of this gene in 60 tumor and normal DNA samples obtained from 43 pancreatic cancer patients treated with gemcitabine. Genotype-phenotype association studies were performed using the SNPs identified during *FKBP5* resequencing, and the phenotypes included overall survival and *FKBP5* gene expression in tumor samples. Functional genomic studies suggested that rs73748206 might contribute to gemcitabine treatment response by increasing *FKBP5* expression through increased binding to glucocorticoid receptor (GR), a known regulator of *FKBP5* expression [12], [13]. This comprehensive *FKBP5* pharmacogenomic study provides enhanced understanding of the role of inheritance in variation in gemcitabine response in the treatment of pancreatic cancer.

## Materials and Methods

### Patient Cohort

The samples were all derived from pancreatic cancer patients treated with gemcitabine at the Mayo Clinic, and included samples from 39 tumor and 21 adjacent tissues (normal), with 19 paired tumor and normal samples. Specifically, these samples were from patients with stage two or three adenocarcinoma who underwent Whipple procedure or pancreatectomy (distal or total) performed at the Mayo Clinic from 2000–2010 and from whom frozen tumor and/or adjacent tissue specimens were available for DNA and RNA extraction. Available phenotypic data for this sample set included overall survival and treatment information. In addition, we obtained genome-wide basal mRNA expression data for all of the tumor tissue samples. Lymphoblastoid cell lines (LCLs) were also established from a subset of these patients. Demographic data for the patients are listed in Table S1. Patients enrolled into the study provided written informed consent for participation in the Mayo Clinic Biospecimen Resource for Pancreas Research. Studies were approved by the Mayo Clinic Institutional Review Board.

### *FKBP5* Gene Resequencing

*FKBP5* was resequenced in 60 DNA samples from pancreatic cancer patients by performing Next Generation sequencing using an Illumina Genome Analyzer IIx (Illumina, San Diego, CA) (Table S2). Resequencing covered a 160 kb genomic region on chromosome 6p21 that contained *FKBP5* utilizing methods described previously [14]. Sanger sequencing of *FKBP5* was used for validation purposes and was performed with the same DNA samples. This type of sequencing was performed in the Mayo Molecular Biology Core Facility with an ABI 3730 DNA sequencer (Applied Biosystems, Foster City, CA). However, in this case, resequencing was focused on regions covering exons, exon-intron splice junctions and approximately 1000 bp of 5′- and 3′-flanking regions (FRs) (Table S3). There was sequence concordance for regions resequenced using both methods.

### Expression Array Data

We generated expression array data for our pancreatic cancer patient cohort as described previously [10]. In brief, RNA was extracted from surgically obtained patient samples with the RNeasy mini kit (Qiagen, Valencia, CA). Once the RNA passed quality control using an Agilent 2100 Bioanalyzer (Agilent Technologies, Inc., Santa Clara, CA), it was hybridized to Affymetrix U133 Plus 2.0 GeneChips (Affymetrix, Inc., Santa Clara, CA). This data have been deposited in the GEO database with the accession number GSE16515.

### Statistical Methods

The relationship between individual SNPs and survival was modeled using Cox proportional hazards models. Due to the relatively small size of the sample, tests based on the likelihood ratio statistic, but using empirical distributions of these statistics based on 50,000 permutations of the phenotype were used. Analysis of individual markers can be underpowered for rare markers. Therefore, we employed two different methods to test the association of a set of markers with the phenotypes. First, to test the association of *FKBP5* markers as a whole or in a sub-region of FKBP5 with *FKBP5* expression, the expression probes representing *FKBP5* were averaged, and then this average was dichotomized based on the median. We then employed our novel method, difference in minor allele frequency (DMAF) test, to test the association of a set of markers with this new *FKBP5* expression phenotype. A more detailed description of DMAF can be found in Brisbin et al. (2012) [15]. Sliding windows of 10–50 markers were used to localize the signal within the gene and type I error within a sliding window size was controlled using permutation based methods, which were also described in further detail by Brisbin et al. [15]. To test the association between survival and SNPs, we used a rare variant burden type test [16]. Briefly, the count of variant alleles was summed over all of the *FKBP5* markers for the analysis of FKBP5 as one entity. At the same time, variants were also summed in smaller regions of 10 to 50 SNPs (windows, or sub-regions of FKBP5). These sums of variants were used as predictors in separate Cox proportional hazards models of survival. The effect of the count of variant alleles was tested using likelihood ratio tests, and permutation based methods were used to control type I error for a given window size [15].

### Cell Culture and Transfections

To create variant constructs for the two nonsynonymous cSNPs, Thr(17)Ala and Glu(383)Leu, that were identified during the resequencing of *FKBP5*, site-directed mutagenesis was employed, as described previously [17], using primers listed in Table S4A. HEK293T cells (ATCC) were used to perform transfections with the pIRES-GFP/Flag empty vector (EV), WT, and variant constructs using the Lipofectamine2000 protocol (Life Technology, Grand Island, NY). GFP signal values were used to correct for possible variation in transfection efficiency.

### Lymphoblastoid Cell Lines and Drug Treatment

Lymphoblastoid cell lines derived from pancreatic cancer patients corresponding to variant or WT genotypes for the rs73748206 SNP were cultured in RPMI plus 15% FBS media, and were used to perform expression analysis of *FKBP5* and *GR*. Assays were performed in triplicate at each drug concentration in 6-well plates at a density of 1×10^6^ cells per well. The cells were treated with gemcitabine (Eli Lilly) (10 mmol/L, 0.1 mmol/L, 0.1 umol/L) or dexamethasone (Steraloids, Newport, RI) (500 nM) for 24 h and mRNA was extracted for the analysis (QuickRNA MiniPrep, Zymo Research, Irvine, CA).

### Real-time Quantitative RT-PCR

Quantitative reverse transcription-PCR (QRT-PCR) was performed with the 1-step Brilliant SYBR Green QRT-PCR master mix kit (Stratagene, La Jolla, CA). Primers were purchased from Qiagen, and all experiments were performed in triplicate with GFP or β-actin as an internal control.

### Reporter Gene Assays

Regions surrounding the SNPs (350–600 bp; depending on the SNP location) were amplified using the PCR and cloned into the pGL3 promoter vector (Promega, Madison, WI). Primer sequences are listed in Table S4B. DNA sequences were verified by Sanger sequencing. Specifically, Su86 and HupT3 cells lines, seeded at 1.5×10^5^ cells/well (6-well plates), were transfected with WT, variant or vector constructs together with a pRL-TK DNA construct encoding *Renilla* luciferase as a control for transfection efficiency. Luciferase activity was measured using a dual luciferase assay (Promega, Madison, WI) with a TD-20/20 Luminometer (Turner Designs, Sunnyvale, CA). Results were expressed as the ratio of firefly luciferase to Renilla luciferase light units. All values were expressed as a percentage of the pGL3 promoter construct activity.

### Electrophoresis Mobility Shift Assay (EMSA)

EMS assays were performed with nuclear extracts from two pancreatic cancer cell lines, Su86 and HupT3 (ATCC), using the LightShiftTM Chemiluminescent EMSA Kit (Pierce, Rockford, IL), as described previously [17]. WT and variant sequences for rs73748206 probes are listed in Table S4C. For competition assays, a 400-fold excess of unlabeled probe was added to the reaction mixture. For the supershift assay, glucocorticoid receptor antibody (GR Ab; Santa Cruz, CA) was added to the reaction mixture.

### Chromatin Immunoprecipitation Assay (ChIP)

ChIP assays were performed using the Imprint Ultra ChIP kit (Sigma-Aldrich, St. Louis, MO) and were performed according to the manufacturer instructions using an antibody against GR or rabbit IgG (Santa Cruz, CA) as a control. Quantitative polymerase chain reaction (Q-PCR) was used to determine the ChIP assay results.

## Results

### Introduction

**Figure 1:**
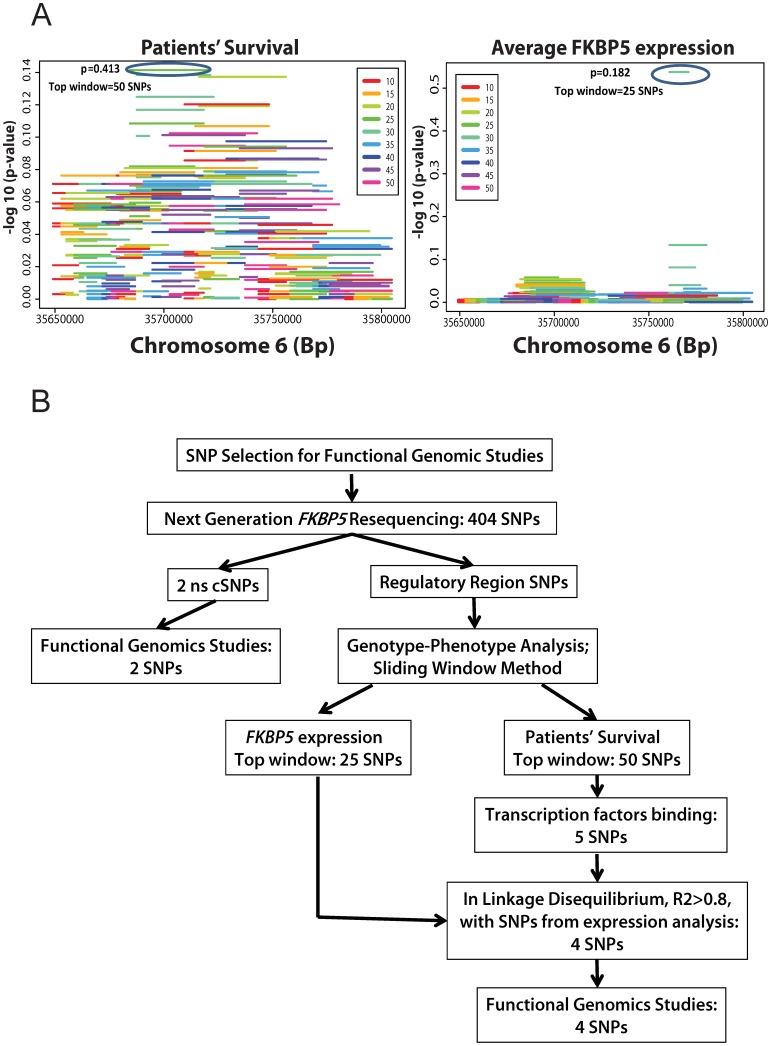
SNP selection for functional genomic studies. (A) Association of patients’ survival (left panel) and *FKBP5* expression (right panel) with groups of *FKBP5* SNPs using Difference in Minor Allele Frequency test. These SNPs are grouped into 10–50 SNPs/group and are arranged based on their chromosome localization. Color indicates different length of SNP window. (B) Flow chart demonstrates the selection criteria of SNPs for the functional characterization studies. Abbreviations: SNP, single nucleotide polymorphism; ns cSNP, non-synonymous coding SNP; DMAF, Difference in Minor Allele Frequency.

We studied the role of genetic variation in *FKBP5* and its effect on *FKBP5* gene transcriptional regulation as well as response to gemcitabine in the treatment of pancreatic cancer. We first focused on the identification of genetic variants in *FKBP5* by the use of Next Generation sequencing of this gene in 60 samples from tumor and adjacent normal tissues that were derived from 43 patients (patient demographic data are listed in Table S1; gene resequencing results are listed in Table S2). We then performed an association study with overall survival and *FKBP5* expression to identify candidate SNPs that might be associated with patient overall survival (OS) or variation in *FKBP5* expression in response to chemotherapy (Figure 1). Finally, functional genomic studies were performed for the non-synonymous cSNPs in addition to the regulatory SNPs selected from the association analysis in an attempt to understand mechanisms by which these SNPs might influence FKPB51 function, and in turn, response to gemcitabine in the treatment of pancreatic cancer.

### *FKBP5* Gene Resequencing

To identify genetic variation in the *FKBP5* gene, we performed Next Generation sequencing of DNA derived from 60 pancreatic tumor and normal tissue samples obtained from pancreatic cancer patients by sequencing an area of 160 kb of DNA on chromosome 6p21. We found 404 SNPs, with 326 having a minor allele frequency (MAF) greater than 1% (Table S2), including 2 non-synonymous coding SNPs (ns cSNPs), Thr(17)Ala and Gln(383)Leu. Thirty two SNPs were novel as compared to the “1000 Genomes Project” (Phase 1 data, [18]) and/or dbSNP data. We also identified three novel insertions/deletions (indels) (Table S5) as compared to our previous *FKBP5* resequencing results utilizing a “Human Variation Panel” sample set involving DNA from 288 subjects of three ethnic groups [19]. Sanger sequencing identified 23 SNPs and all of them were in concordance with the results of the Next Generation sequencing (Table S3).

### Association Analysis

While observing an overall survival (OS) of pancreatic cancer patients from whom DNA was obtained for sequencing of *FKBP5*, the ns cSNP, Thr(17)Ala, a SNP that was in perfect linkage disequilibrium, LD, (LD = 1) with two other regulatory SNPs, was only present in the individual with the shortest survival time of all individuals in this set of samples. This observation provided anecdotal evidence of interest. This SNP had a MAF<1%, indicating that it might be a rare, private mutation identified in this particular patient, although this same SNP was observed in the “1000 Genomes” project [18] with the same MAF<1%.

Next, we looked at the entirety of *FKBP5*, and sub-regions within the gene (via sliding windows), to aggregate signals from both rare and common SNPs with survival and FKBP5 expression. For survival, there were 50 SNPs in the top window for this association (in tumor samples only) with p = 0.413, as illustrated in Figure 1 (top window SNPs listed in Table S6). We also performed sliding window analysis of resequenced SNPs for their association with *FKPB5* expression levels for the same samples. Since *FKBP5* has three probe sets represented in the Affymetrix expression array, 224856_at, 224840_at and 204560_at, we decided to use the top window associated with average expression levels derived from all three *FKBP5* probe sets. The top window that was associated with *FKPB5* expression contained 25 SNPs with a p-value = 0.182, as shown in Figure 1A (top window SNPs listed in Table S6). To gain the mechanistic insights of these variants, we pursued them with functional genomic studies.

### Functional Genomic Studies

#### Ns cSNPs: Ala^17^ and Leu^383^

We began our functional genomic studies with characterization of SNPs found in the coding regions. In our sample set, two ns cSNPs were identified in both tumor and adjacent tissue samples with MAFs for both SNPs <3%. Even though Ala^17^ was observed only in the patient with the shortest survival time, we studied both of the ns cSNPs since they might alter the level and function of the encoded enzyme, as has been shown by our laboratory previously [20]. Expression constructs were created for WT and variant sequences of the ns cSNPs and overexpressed in HEK293T as well as Su86 and Miapaca2 cell lines (ATCC). Quantitative Western blot analysis was completed to determine protein levels, followed by determining mRNA levels with QRT-PCR (Figure S1A). Additionally, we performed gemcitabine cytotoxicity studies using cells overexpressing WT and variant constructs to see if these FKBP51 variant allozymes would affect response to drug treatment (Figure S1A **)**. There were no statistically significant differences between the FKBP51 WT and variant allozymes in the results of any of the functional assays.

#### SNPs in regulatory regions

Next, we focused on characterization of the SNPs in the regulatory regions, since these SNPs might affect expression of *FKBP5* which is critical for its effect on gemcitabine response [7]. Based on the sliding window association analysis, there were 50 SNPs in the top window for association with survival, as illustrated in Figure 1A. Even though the p-value for this association was not significant, which could be due to the low number of the patient samples we had available for the analysis, we still decided to examine this group of SNPs since they might help us understand the mechanisms involved in transcriptional regulation of *FKBP5*. Among the 50 SNPs, except for one ns cSNP (Thr(17)Ala), all 49 SNPs were located in regulatory regions of the gene. First, we determined if any of these 49 SNPs were in regions of transcription factor (TF) binding by annotating them using the Human Genome Browser (http://genome.ucsc.edu). Five [(rs73746499, rs116796504, 35716217 (genomic location of a novel SNP for which an rs# has not been assigned), rs73748206, rs148128369)] of the 49 SNPs were in a region predicted to bind TFs, as illustrated in the flow chart in Figure 1B. SNPs rs73746499 and rs73748206 were in LD, R^2^ = 0.82, and both had MAFs of 4%. SNPs rs116796504, 35716217, and rs148128369 had MAFs<1%. Since the expression of *FKBP5* regulates cell chemosensitivity [7] and, therefore, it can affect patients survival, we next checked if any of these five SNPs selected from the survival analysis were in linkage disequilibrium (LD) with the SNPs in the top window from the sliding window analysis of *FKBP5* expression (Figure S2). Four SNPs (rs73746499, rs116796504, rs73748206, rs148128369) were in LD with the SNPs in the top window from the expression analysis (R^2^>0.8). Therefore, those four SNPs were selected for further functional characterization studies (Table 1).

**Table 1:** SNPs selected for functional characterization studies.

SNPs selected for functional genomic studies			
rs#	Location	MAF	TF
rs148128369	35720889/Intron 1	<1%	NFKB
rs73748206	35715933/Intron 2	4%	GR
rs73746499	35686829/Intron 5	4%	GR
rs116796504	35686808/Intron 5	<1%	GR

Abbreviations: MAF, minor allele frequency; TF, transcription factor; GR, glucocorticoid receptor.

#### Functional characterization of regulatory SNPs

**Figure 2:**
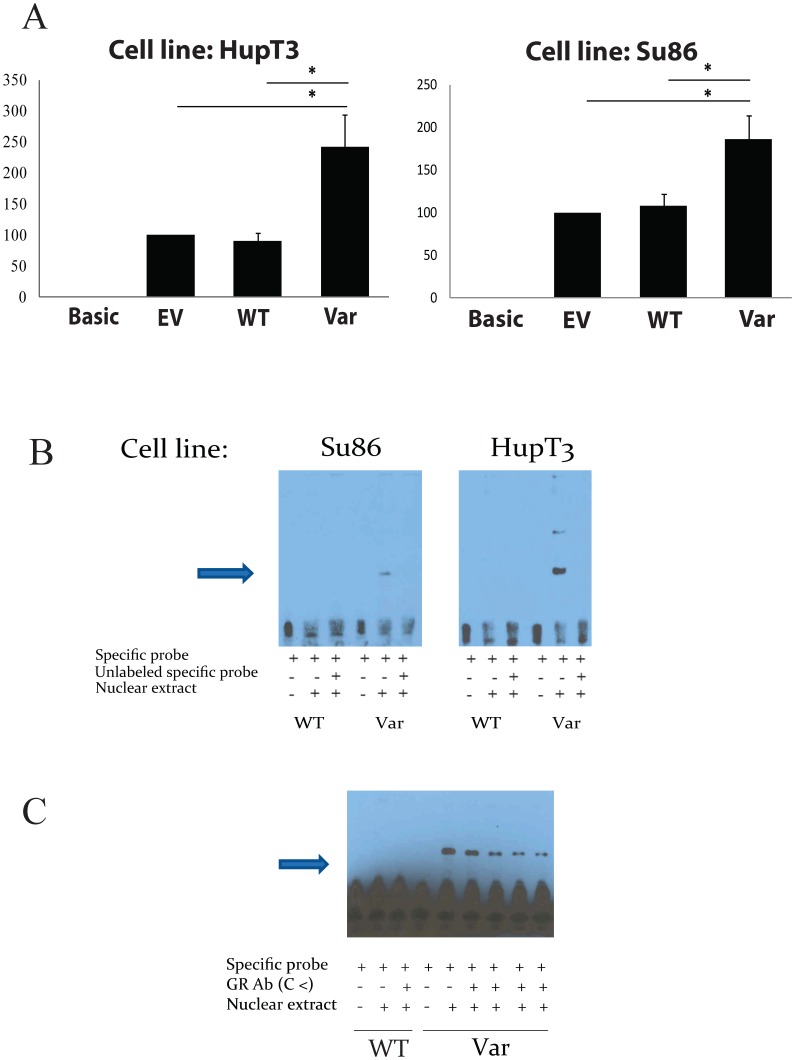
Functional characterization of the rs73748206 using pancreatic cancer cell lines. (A) Reporter Gene Assay performed in HupT3 and Su86 cells, using Basic and pGL3 promotor constructs: empty vector (EV), wild type (WT), and variant (Var). Error bars for each construct represent the average of relative luciferase activity calculated as a % of the pGL3-Promoter construct activity obtained during 6 independent transfections (means ± S.E.M). * represent T-test p-values for comparing values of Var and WT, and Var and EV activity. (B) EMS assays with nuclear extract derived from Su86 and HupT3 cell lines. An arrow indicates the band that was observed with the variant but not the WT sequences. (C) Supershift assay. Increasing concentrations of anti- glucocorticoid receptor antibody were incubated with both, WT and Variant sequences, and Su86 nuclear extract.

We performed a series of experiments to assess the possible functional significance of the selected SNPs. We first performed a reporter gene assay to determine the effect of these four SNPs on transcriptional activity using two human pancreatic cancer cell lines, Su86 and HupT3, both of which express *FKBP5*. The regions containing the rs73746499 and rs148128369 SNPs did not show a significant change in luciferase activity (data not shown). Transcriptional activity was significantly decreased with the reporter gene construct containing the region harboring the rs116796504 SNP as compared to the luciferase promoter control in both cell lines (p-value <0.05). However, only in HupT3 cells but not in Su86 cells, there was a statistically significant difference between WT and the Variant construct (p-value <0.05). On the other hand, the region containing the variant sequence for rs73748206 resulted in a more than two fold induction in luciferase activity as compared to the WT and the control (Figure 2A) in both pancreatic cancer cell lines, Su86 and HupT3, (p-value <0.05). Based on these initial results we decided to focus our further functional characterization on the rs73748206 SNP.

We next performed electromobility shift assays (EMSA) using nuclear extract from Su86 and HupT3. Figure 2B shows that incubation with the nuclear extract resulted in complex formation in the presence of variant, but not WT oligonucleotides for rs73748206 in both cell lines. Next, we wanted to see if co-incubation of the oligonucleotides that showed a differential protein-DNA binding pattern with antibodies against the transcription factor (TF), predicted by TransFast database, would reduce the intensity of the band. For the rs73748206 SNP, addition of increased concentrations of GR antibody in the presence of the variant oligonucleotides resulted in the gradual disappearance of the initially observed band (Figure 2C). This result suggested that GR antibody can competitively bind to the transcription factor complex present in the nuclear extract, and that this transcription factor complex including GR is responsible for binding to the DNA region surrounding the rs73748206.

**Figure 3:**
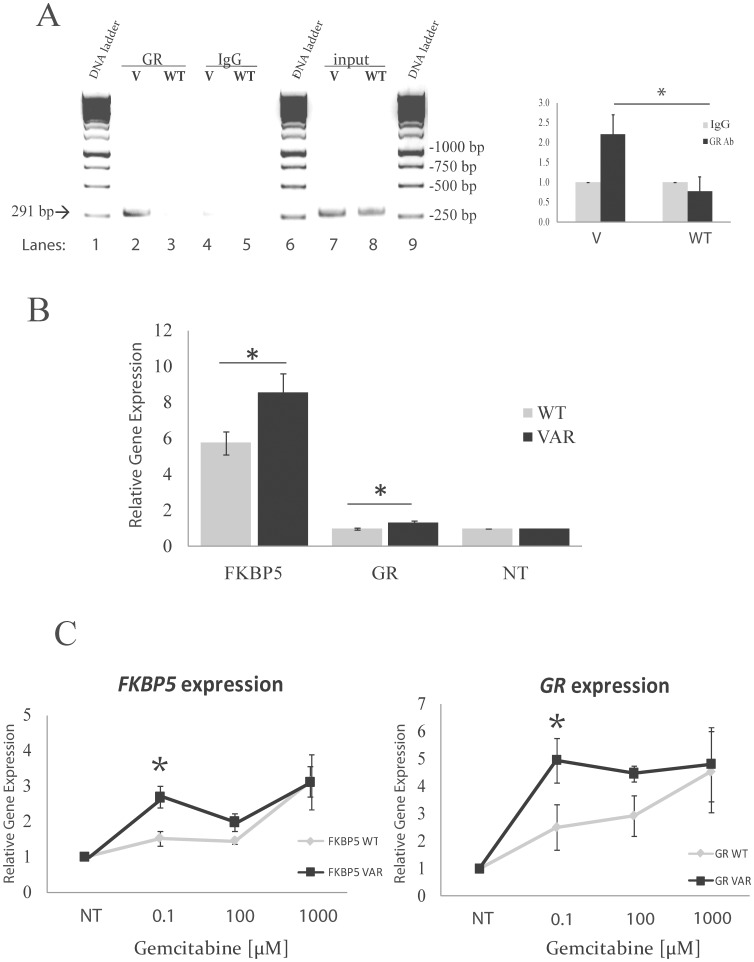
Functional characterization of the rs73748206 using pancreatic cancer patients’ lymphoblastoid cell lines (LCLs). (A) ChIP assay using lymphoblastoid cells with known genotype for the rs73748206 single nucleotide polymorphism (SNP). Left panel indicates a picture of gel electrophoresis. Lanes 1, 6, 9 correspond to DNA ladders. Lanes 2 to 5 are polymerase chain reaction (PCR) products from DNA that was bound to human GR (glucocorticoid receptor) antibody (GR) (lanes 2 and 3) or immunoglobulin G (IgG) control (lanes 4 and 5). The inputs for the variant (V) (lane 7) and wild type (WT) (lane 8) were PCR amplification products of pools of sheared DNA from the entire genome. Right panel is a ChIP qPCR analysis as % of input, shown as fold change relative to IgG. (B) SNP-related differences in relative FKBP5 and GR gene expression after exposure to dexamethasone for 24 h in variant (VAR) and WT cells; (*) p-value *<*0.05. (C) SNP-related differences in *FKBP5* and *GR* expression (left and right panels, respectively), and gemcitabine response in variant (VAR), n = 1, and WT, n = 3, cell lines. Data is representative of three independent experiments (mean +/− SEM); (*) p-value <0.05.

Finally, we took advantage of LCL derived from the same 43 patients to determine the effect of rs73748206 *in vivo*. Using these cell lines, we could perform a series of experiments based on *FKBP5* rs73748206 genotypes, since this particular variant was found in both tumor and normal tissues. We first confirmed the sequence by sequencing a 689 bp region surrounding the SNP using DNA from the LCLs that we had available for the variant (n = 1) and the WT (n = 3). In this sample set, we only had a heterozygous cell line for the rs73748206 SNP. Therefore we only used the heterozygous cell line for the following experiments. It is already known that *FKBP5* expression is regulated by GR and that GR sensitivity can also be regulated by FKBP51 [21]. In the next series of experiments, we wanted to determine whether GR binds to rs73748206 using these cell lines with the naturally occurred SNP. We first performed a chromatin immunoprecipitation (ChIP) assay by utilizing cell lines homozygous for rs73748206 WT or heterozygote for the variant that were pretreated with 500 nM dexamethasone. There was a two-fold increase in binding of GR in heterozygous as compared to WT cells (Figure 3A). We then determined the effect of different genotypes of this SNP on *FKBP5* expression. There was a significant change in expression of *FKBP5* in the heterozygous cells as compared to WT (Figure 3B). To test whether gemcitabine treatment might have an effect on *FKBP5* expression in LCLs and if so, whether there was a SNP-dependent effect on this regulation, we treated WT and heterozygous cells with gemcitabine and determined the *FKBP5* expression levels. We found that the expression of *FKBP5* was significantly altered in a SNP-dependent fashion with increased concentrations of gemcitabine (Figure 3C). Interestingly, we also found that GR expression levels were also altered in a SNP and gemcitabine dosage-dependent fashion (Figure 3C). Particularly, there was a significant increase (p-value <0.05) in expression of *FKBP5* and *GR* in cells with the heterozygous genotype at around 0.1 µM of gemcitabine, a concentration that is close to gemcitabine IC_50_ value in these cell lines [7]. These results suggested a feedback loop between FKBP5 and GR regulation in a SNP-dependent fashion. Results for the functional characterization of rs73748206 were consistent between *in vitro* and *in vivo* studies, which would suggest that this particular variant SNP might contribute to gemcitabine treatment response.

## Discussion

Pancreatic cancer treatment remains challenging. Therefore, novel approaches to individualize treatment are needed to fight this fatal disease. There have been several DNA sequencing studies of pancreatic tumor that were designed to define the predominant mutations in this disease [22], [23]. All of those studies were performed with a small number of pancreatic tumor samples (n<25). That is most likely due to the fact that pancreatic cancer is not diagnosed until it is already in its late stage or is metastatic, thus precluding surgery and resecting the tumor. Because of a limited number of treatment options for pancreatic cancer patients, novel insights into not only the pathogenesis of this disease but also into its therapy are needed.

After Burris et al [4] demonstrated that gemcitabine is a superior drug as compared to fluorouracil (5-FU) in advanced pancreatic cancer, gemcitabine became the standard of treatment for this lethal disease. We previously have shown that variation in *FKBP5* gene expression is the contributor to the variation in gemcitabine response due to the role of FKBP51 in regulation of the Akt pathway [7], [10], [24]. In the present study, we set out to comprehensively study how *FKBP5* genetic polymorphisms might play a role in gemcitabine response and, ultimately, overall survival of pancreatic cancer patients. Therefore, we focused on functional characterization of genetic variation in *FKBP5* that we identified through Next Generation DNA sequencing (Table S2). We first performed a genotype-phenotype association analysis using a sliding window methodology (Figure 1A) to identify and then to systematically study the top selected genetic variants and their role in response to gemcitabine treatment. Interestingly, when we examined overall survival of the patients, one of the two ns cSNPs, Thr(17)Ala, that we identified during the resequencing study was found in the individual with the poorest survival after drug treatment. However, we did not observe any significant alteration as a result of this allozyme during our functional studies (Figure S1). This same SNP was observed in the “1000 Genomes Project” [18]. However, it was not included in the Catalog of Somatic Mutations in Cancer (COSMIC), most likely due to the low MAF of this SNP (<1%), and a low number of samples in this database (n = 23) [22].

Next, we focused on the functional characterization of SNPs within regulatory regions of *FKBP5*. We performed a sliding window analysis, a method that allowed us to consider not only common but also rare variants together (Figure 1A) [15]. Despite the fact that the p-values for association of *FKBP5* SNPs with patient survival or *FKBP5* gene expression were not significant, this approach helped us to identify a novel functional SNP, rs73748206, which influenced *FKBP5* expression through interaction with the glucocorticoid receptor (Figures 1, 2 and 3). This SNP is located in Intron 2 of *FKBP5* and the “C (G)” genotype is conserved across species (www.ensembl.org). It has a 4% MAF for “T (A)” which is confirmed by “1000 Genomes Project” data. This SNP is in LD (R^2^ = 1) with rs73746499, a SNP that we originally had chosen to functionally characterize, but did not observe any significant changes in our initial studies of that SNP. Rs73748206 is located within a GR binding site and also within DNaseI hypersensitivity clusters (annotated at http://genome.ucsc.edu), indicating that this area may be transcriptionally active, which was confirmed in our functional studies (Figures 2 and 3). There are no predicted methylation sites within the region containing rs73748206 (annotated at http://genome.ucsc.edu). GR is a transcription factor known to modulate *FKBP5* gene expression [12], [13] as well as its own expression [25], [26], [27], [28]. Conversely, FKB51 protein negatively regulates GR sensitivity to glucocorticoids [29]. In our preliminary studies, we noticed that knock-down of *FKBP5* expression in pancreatic cancer cells can contribute to decreased *GR* expression (Figure S3A). In addition, when we treated pancreatic cancer cells with gemcitabine, the expression of *FKBP5* as well as *GR* was upregulated in a dose dependent manner (Figure S3B), even when *GR* expression was initially knocked-down in these cells (Figure S3C). These preliminary experiments suggested that *FKBP5* might also contribute to the regulation of *GR* expression. This relationship might be due to the fact that FKBP5 regulates GR activity [21], [30], [31] which influences GR function as a transcription factor that can regulate both *FKBP5* gene expression and the expression of GR itself [26], [27], [28]. In turn, if there is an increase in GR expression in the cell, that might increase *FKBP5* gene expression. This phenomenon could possibly explain the effect of rs73748206 on the regulation of *FKBP5* and *GR* as well as its effect on gemcitabine response in a SNP-dependent manner.

There has been an increased interest in FKBP51 over the past decade, especially in terms of its implications in psychiatry or psychological disorders (reviewed in [21]). This is mainly due to its chaperone role in a complex with HSP90 and GR and its role in responsiveness to glucocorticoids (reviewed in [32]). Our laboratory previously reported a novel function of FKBP51 and its role in cancer in response to chemotherapy, particularly treatment with cytidine analogues, gemcitabine and cytarabine [7]. *FKBP5* SNPs have already been implicated in regulating treatment response in cancer, such as acute myeloid leukemia [33]. In the study by Mitra et al. in which patients were treated with cytarabine, another cytidine analogue, it was shown that an intronic SNP, rs3798346, was significantly associated with event free and overall survival, indicating that *FKBP5* polymorphisms might contribute to drug treatment response.

In summary, the present study provides additional evidence suggesting that SNPs in *FKBP5* might contribute to gemcitabine response in the treatment of pancreatic cancer. Although our study needs to be validated in a large cohort, it indicates that rs73748206 could potentially be a novel biomarker to predict patient response to gemcitabine therapy and represents a step toward the individualized chemotherapy of pancreatic cancer.

## Supporting Information

Figure S1 Functional characterization of FKBP5 allozymes. (A) Left panel. Quantitative Western blot analysis of FKBP5 Allozymes: WT, Thr(17)Ala, and Gln(383)Leu expression constructs in HEK293T cells performed with anti-Flag antibody; Right panel. qRT-PCR analysis of FKBP5 WT, Thr(17)Ala, and Gln(383)Leu mRNA expression levels in HEK293T cells. EV = empty vector. All values are representative of three independent experiments (mean+SEM.). (B) Cytotoxicity assays. Pancreatic cancer cell lines, Miapaca2 and Su86, were transfected with FKBP5 WT, Thr(17)Ala, and Gln(383)Leu expression constructs and treated with gemcitabine. Points show mean values for three independent experiments; error bars represent standard error of the mean (SEM). (PDF)

Figure S2 Linkage disequilibrium (LD) plot of selected *FKBP5* SNPs. SNPs were selected based on the sliding window analysis (see Figure 1). The extent of linkage disequilibrium for the top SNPs from *FKBP5* expression analysis and 5 SNPs binding to transcription factors (TF) from survival analysis, shown as pairwise R^2^- values, is depicted graphically. (PDF)

Figure S3 Correlation between *FKBP5* and *GR* gene expression levels. (A) Relative *FKBP5* and *GR* gene expression after *FKBP5* knock-down in pancreatic cancer cells, 48 h after *FKBP5* siRNA treatment. (B) Relative FKBP5 or GR gene expression (left or right panels, respectively) in Su86 or HupT3 cell lines (upper or lower panels, respectively) exposed to increased gemcitabine concentrations as compared to no treatment (NT). (C) Relative expression of GR 96 h after GR knock-down in Su86 cells (*left panel*). Relative FKBP5 expression after GR knock-down in Su86 cells treated with increasing concentration of gemcitabine over 72 h (*right panel*). *Columns* – mean average of three (or two) independent experiments*; Bars –* SEM. (PDF)

Table S1 Demographic information for pancreatic cancer patients used in this study. (PDF)

Table S2 List of Next Generation resequenced FKBP5 SNPs. (PDF)

Table S3 Human FKBP5 gene Sanger resequencing. (PDF)

Table S4 Oligonucleotide sequences for site-directed mutagenesis and reporter gene assay, and EMSA probes. (PDF)

Table S5 Novel insertions/deletions (Indels) detected by Next Generation resequencing analysis of pancreatic patients DNA samples. (PDF)

Table S6 List of top window SNPs from sliding window association analysis with average FKBP5expression and overall survival. In bold are the SNPs selected for functional characterization. (PDF)

## References

[1] Siegel R, Naishadham D, Jemal A (2012) Cancer statistics, 2012. CA: A Cancer Journal for Clinicians 62: 10–29. 22237781 10.3322/caac.20138

[2] Institute NC Surveillance epidemiology and end results. 2012 [cited; Available from: http://seer.cancer.gov/csr/1975_2009_pops09/index.html. Accessed 2013 July 5].

[3] Ghaneh P, Costello E, Neoptolemos JP (2007) Biology and management of pancreatic cancer. Gut 56: 1134–1152. 17625148 10.1136/gut.2006.103333 PMC1955499

[4] Burris HA, Moore MJ, Andersen J, Green MR, Rothenberg ML, et al (1997) Improvements in survival and clinical benefit with gemcitabine as first-line therapy for patients with advanced pancreas cancer: a randomized trial. J Clin Oncol 15: 2403–2413. 9196156 10.1200/JCO.1997.15.6.2403

[5] Mini E, Nobili S, Caciagli B, Landini I, Mazzei T (2006) Cellular pharmacology of gemcitabine. Ann Oncol 17: v7–v12. 16807468 10.1093/annonc/mdj941

[6] Gilbert JA, Salavaggione OE, Ji Y, Pelleymounter LL, Eckloff BW, et al (2006) Gemcitabine pharmacogenomics: cytidine deaminase and deoxycytidylate deaminase gene resequencing and functional genomics. Clin Cancer Res 12: 1794–1803. 16551864 10.1158/1078-0432.CCR-05-1969

[7] Li L, Fridley B, Kalari K, Jenkins G, Batzler A, et al (2008) Gemcitabine and cytosine arabinoside cytotoxicity: association with lymphoblastoid cell expression. Cancer Res 68: 7050–7058. 18757419 10.1158/0008-5472.CAN-08-0405 PMC2562356

[8] Fischer G, Bang H, Mech C (1984) [Determination of enzymatic catalysis for the cis-trans-isomerization of peptide binding in proline-containing peptides]. [Article in German]. Biomed Biochim Acta 43: 1101–1111. 6395866

[9] Nair SC, Rimerman RA, Toran EJ, Chen S, Prapapanich V, et al (1997) Molecular cloning of human FKBP51 and comparisons of immunophilin interactions with Hsp90 and progesterone receptor. Mol Cell Biol 17: 594–603. 9001212 10.1128/mcb.17.2.594 PMC231784

[10] Pei H, Li L, Fridley BL, Jenkins GD, Kalari KR, et al (2009) FKBP51 affects cancer cell response to chemotherapy by negatively regulating Akt. Cancer Cell 16: 259–266. 19732725 10.1016/j.ccr.2009.07.016 PMC2755578

[11] Hou J, Wang L (2012) FKBP5 as a selection biomarker for gemcitabine and Akt inhibitors in treatment of pancreatic cancer. PLoS One 7: e36252. 22590527 10.1371/journal.pone.0036252 PMC3348935

[12] Hubler TR, Scammell JG (2004) Intronic hormone response elements mediate regulation of FKBP5 by progestins and glucocorticoids. Cell Stress Chaperones 9: 243–252. 15544162 10.1379/CSC-32R.1 PMC1065283

[13] Baughman G, Wiederrecht GJ, Chang F, Martin MM, Bourgeois S (1997) Tissue distribution and abundance of human FKBP51, an FK506-binding protein that can mediate calcineurin inhibition. Biochem Biophys Res Commun 232: 437–443. 9125197 10.1006/bbrc.1997.6307

[14] Pelleymounter LL, Moon I, Johnson JA, Laederach A, Halvorsen M, et al (2011) A novel application of pattern recognition for accurate SNP and indel discovery from high-throughput data: Targeted resequencing of the glucocorticoid receptor co-chaperone FKBP5 in a Caucasian population. Mol Genet Metab 104: 457–469. 21917492 10.1016/j.ymgme.2011.08.019 PMC3224211

[15] Brisbin A, Jenkins GD, Ellsworth K, Wang L, Fridley BL (2012) Localization of association signal from risk and protective variants in sequencing studies. Front Genet 3: 173. 22973297 10.3389/fgene.2012.00173 PMC3434438

[16] Li B, Leal SM (2008) Methods for detecting associations with rare variants for common diseases: application to analysis of sequence data. Am J Hum Genet 83: 311–321. 18691683 10.1016/j.ajhg.2008.06.024 PMC2842185

[17] Wang L, Ellsworth KA, Moon I, Pelleymounter LL, Eckloff BW, et al (2010) Functional genetic polymorphisms in the aromatase gene CYP19 vary the response of breast cancer patients to neoadjuvant therapy with aromatase inhibitors. Cancer Res 70: 319–328. 20048079 10.1158/0008-5472.CAN-09-3224 PMC2804935

[18] Genomes Project Consortium, Abecasis GR, Altshuler D, Auton A, Brooks LD, et al (2010) A map of human genome variation from population-scale sequencing. Nature 467: 1061–1073. 20981092 10.1038/nature09534 PMC3042601

[19] Ellsworth K, Moon I, Eckloff BW, Fridley BL, Jenkins GD, et al (2013) FKBP5 genetic variation: association with selective serotonin reuptake inhibitor treatment outcomes in major depressive disorder. Pharmacogenet Genomics 23: 156–166. 23324805 10.1097/FPC.0b013e32835dc133 PMC3784025

[20] Weinshilboum R, Wang L (2004) Pharmacogenetics: inherited variation in amino acid sequence and altered protein quantity. Clin Pharmacol Ther 75: 253–258. 15080131 10.1016/j.clpt.2003.12.002

[21] Binder EB (2009) The role of FKBP5, a co-chaperone of the glucocorticoid receptor in the pathogenesis and therapy of affective and anxiety disorders. Psychoneuroendocrinology 34 Suppl 1S186–195. 19560279 10.1016/j.psyneuen.2009.05.021

[22] Jones S, Zhang X, Parsons DW, Lin JC-H, Leary RJ, et al (2008) Core signaling pathways in human pancreatic cancers revealed by global genomic analyses. Science 321: 1801–1806. 18772397 10.1126/science.1164368 PMC2848990

[23] Yachida S, Jones S, Bozic I, Antal T, Leary R, et al (2010) Distant metastasis occurs late during the genetic evolution of pancreatic cancer. Nature 467: 1114–1117. 20981102 10.1038/nature09515 PMC3148940

[24] Li L, Fridley BL, Kalari K, Jenkins G, Batzler A, et al (2009) Gemcitabine and arabinosylcytosin pharmacogenomics: genome-wide association and drug response biomarkers. PLoS One 4: e7765. 19898621 10.1371/journal.pone.0007765 PMC2770319

[25] Geng C-D, Schwartz JR, Vedeckis WV (2008) A conserved molecular mechanism is responsible for the auto-up-regulation of glucocorticoid receptor gene promoters. Mol Endocrinol 22: 2624–2642. 18945813 10.1210/me.2008-0157 PMC2626199

[26] Breslin MB, Geng CD, Vedeckis WV (2001) Multiple promoters exist in the human GR gene, one of which is activated by glucocorticoids. Mol Endocrinol 15: 1381–1395. 11463861 10.1210/mend.15.8.0696

[27] Turner JD, Alt SR, Cao L, Vernocchi S, Trifonova S, et al (2010) Transcriptional control of the glucocorticoid receptor: CpG islands, epigenetics and more. Biochem Pharmacol 80: 1860–1868. 20599772 10.1016/j.bcp.2010.06.037

[28] Govindan MV, Pothier F, Leclerc S, Palaniswami R, Xie B (1991) Human glucocorticoid receptor gene promotor-homologous down regulation. J Steroid Biochem Mol Biol 40: 317–323. 1958537 10.1016/0960-0760(91)90197-d

[29] Denny WB, Prapapanich V, Smith DF, Scammell JG (2005) Structure-function analysis of squirrel monkey FK506-binding protein 51, a potent inhibitor of glucocorticoid receptor activity. Endocrinology 146: 3194–3201. 15802496 10.1210/en.2005-0027

[30] Grad I, Picard D (2007) The glucocorticoid responses are shaped by molecular chaperones. Mol Cell Endocrinol 275: 2–12. 17628337 10.1016/j.mce.2007.05.018

[31] Vermeer H, Hendriks-Stegeman BI, van der Burg B, van Buul-Offers SC, Jansen M (2003) Glucocorticoid-induced increase in lymphocytic FKBP51 messenger ribonucleic acid expression: a potential marker for glucocorticoid sensitivity, potency, and bioavailability. J Clin Endocrinol Metab 88: 277–284. 12519866 10.1210/jc.2002-020354

[32] Galigniana NM, Ballmer LT, Toneatto J, Erlejman AG, Lagadari M, et al (2012) Regulation of the glucocorticoid response to stress-related disorders by the Hsp90-binding immunophilin FKBP51. J Neurochem 122: 4–18. 22548329 10.1111/j.1471-4159.2012.07775.x

[33] Mitra AK, Crews K, Pounds S, Cao X, Downing JR, et al (2011) Impact of genetic variation in FKBP5 on clinical response in pediatric acute myeloid leukemia patients: a pilot study. Leukemia 25: 1354–1356. 21483441 10.1038/leu.2011.74 PMC3238383

