# Supplementary figures and images for: Contribution of FKBP5 Genetic Variation to Gemcitabine Treatment and Survival in Pancreatic Adenocarcinoma
Source: PLoS One. 2013 Aug 1;8(8):e70216. doi: 10.1371/journal.pone.0070216 (PMC3731355; doi:10.1371/journal.pone.0070216)

# Figure S1

## A

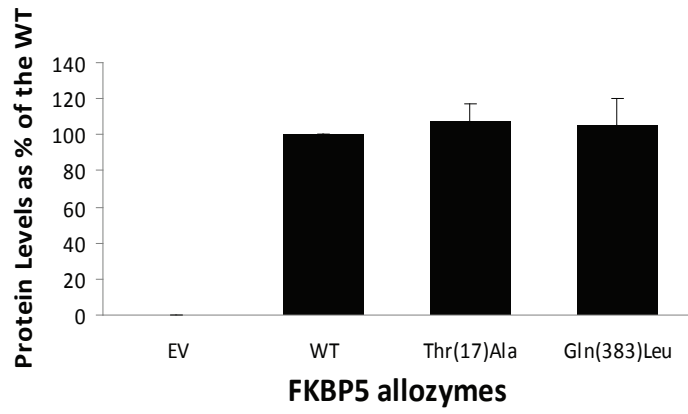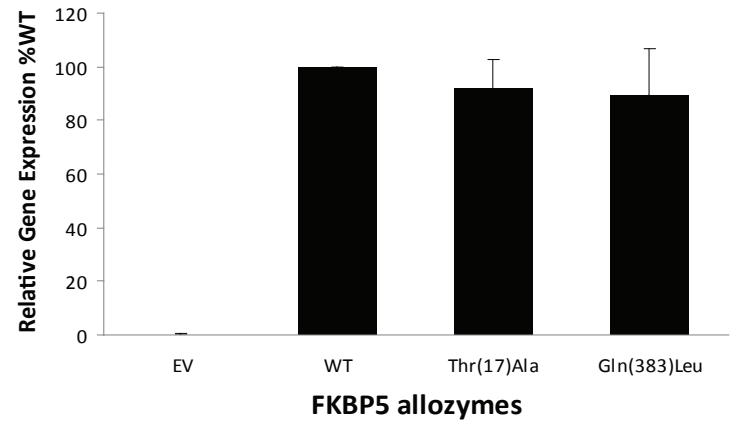

## B

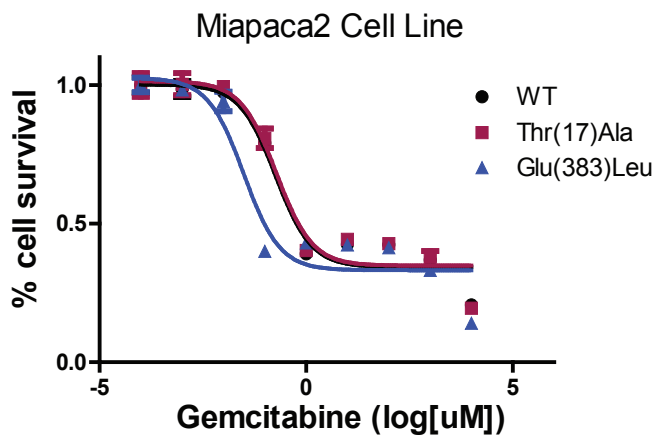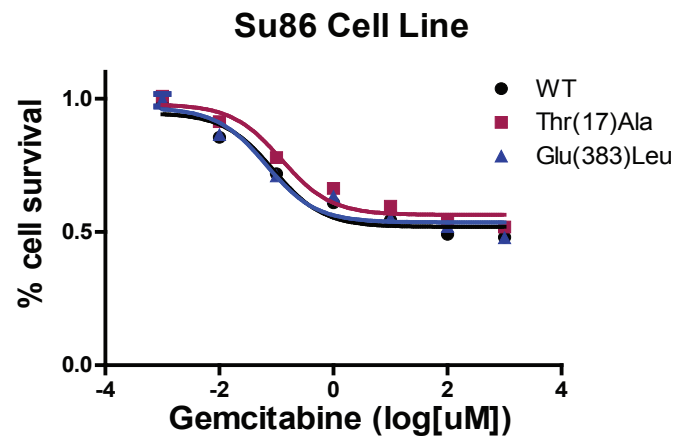

Supplement: Figure S1 — (PDF) [file pone.0070216.s001.pdf]

Figure S2

Patients' Survival SNPs

*FKBP5* expression SNPs

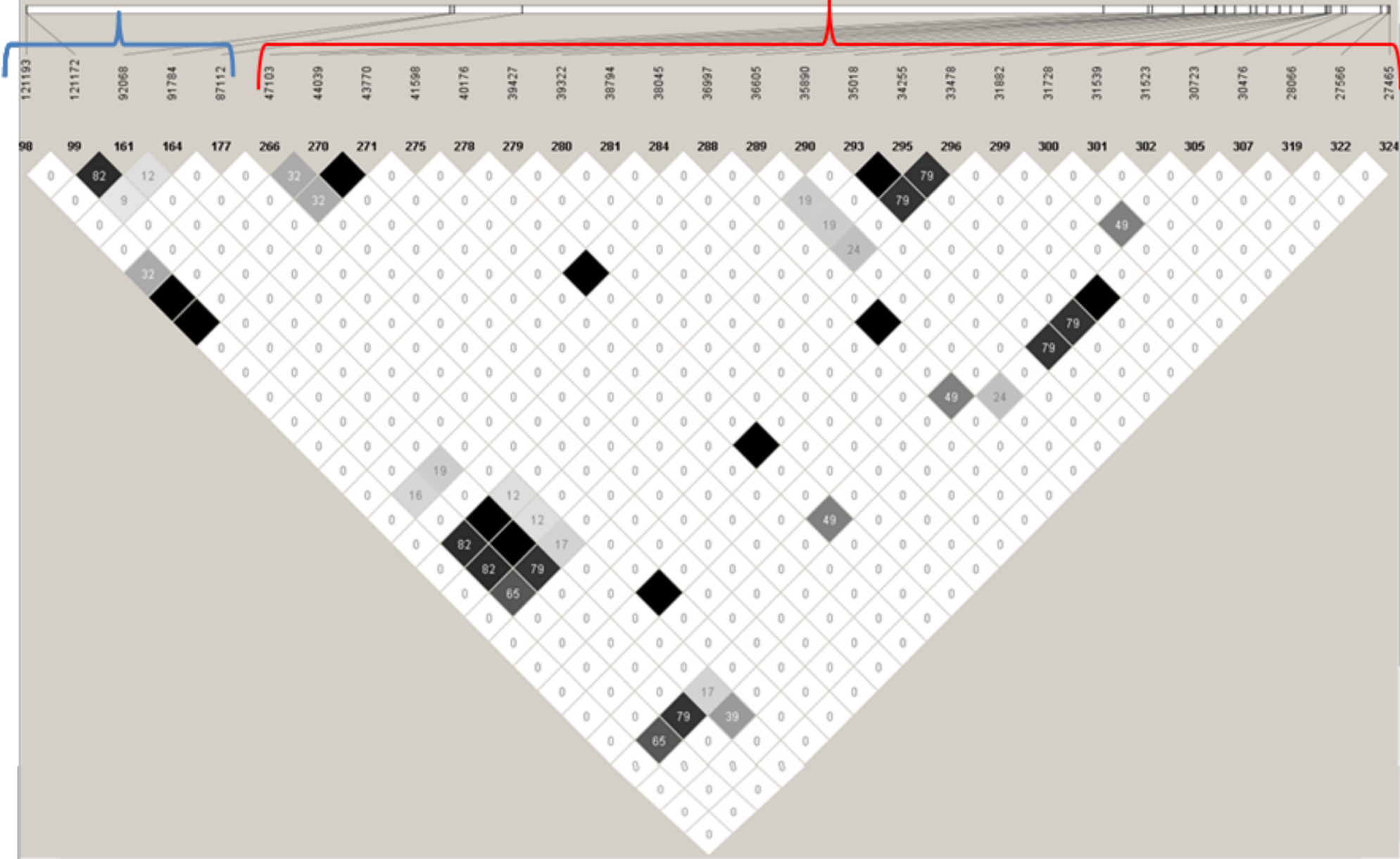

Supplement: Figure S2 — (PDF) [file pone.0070216.s002.pdf]

**Figure S3**

**A**

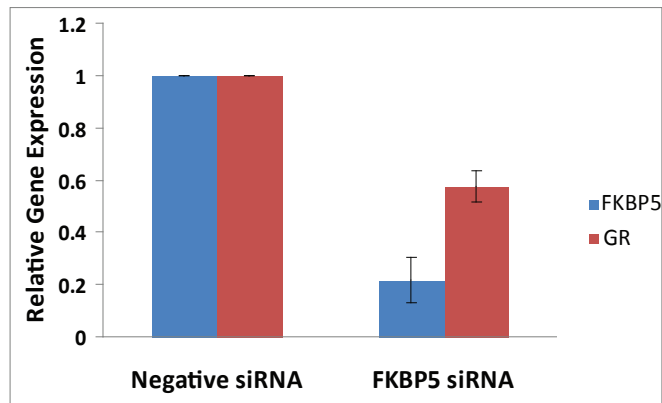

**B**

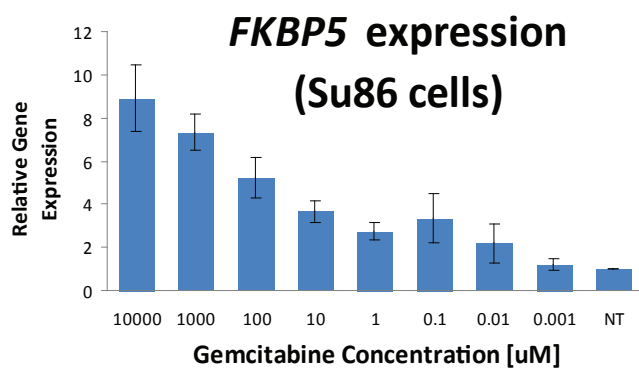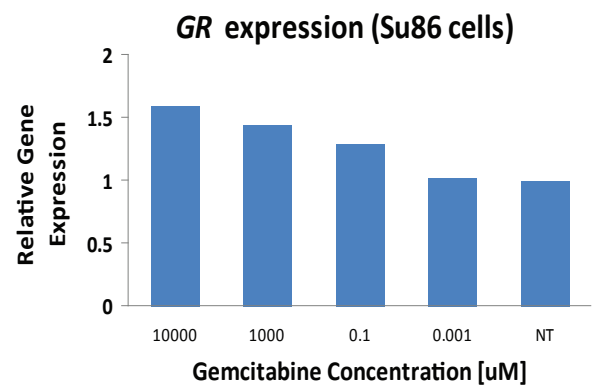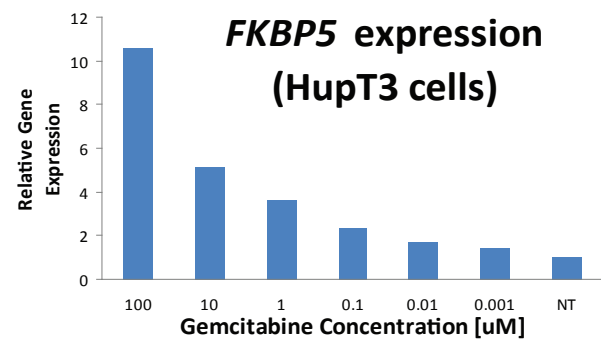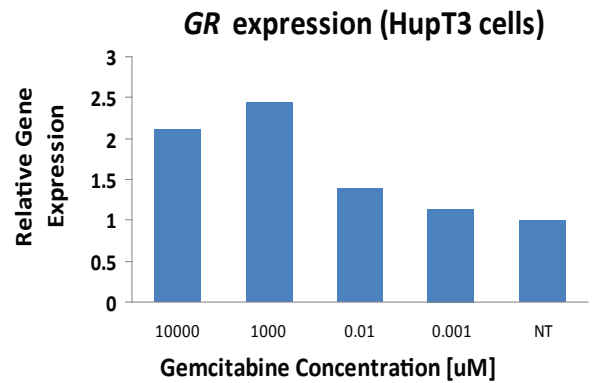

**C**

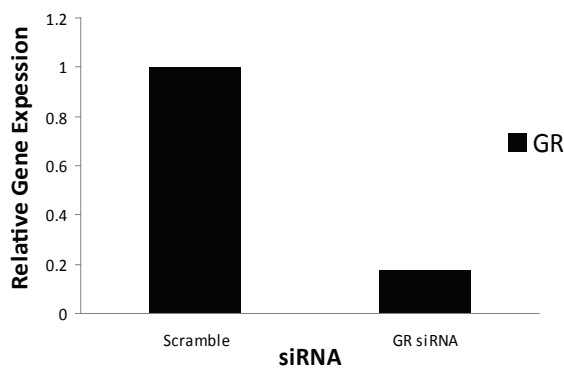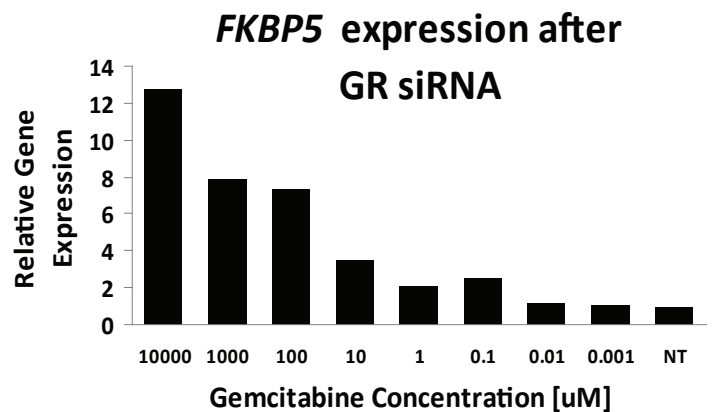

Supplement: Figure S3 — (PDF) [file pone.0070216.s003.pdf]
