# Supplementary material for: Contribution of FKBP5 Genetic Variation to Gemcitabine Treatment and Survival in Pancreatic Adenocarcinoma
Source: PLoS One. 2013 Aug 1;8(8):e70216. doi: 10.1371/journal.pone.0070216 (PMC3731355; doi:10.1371/journal.pone.0070216)
Supplement: Table S1 — (PDF) [file pone.0070216.s004.pdf]

Table S1. Demographic information for pancreatic cancer patients used in this study.

Patient Information	Statistic or group	Value or N(%) Individuals
Age at Pancreas Cancer Diagnosis	n (# missing)	43 (0)
	Mean (SD)	64.91 (10.64)
	Median (min:max)	66.0 (41.0 : 84.0)
	95% CI for mean (t-based)	(61.63, 68.18)
Race	caucasian	42 (97.7)
	multi-racial	1 (2.3)
Gender	Female	16 (37.2)
	Male	27 (62.8)
Smokes	No	22 (53.7)
	Yes	19 (46.3)
Pack Years	n (# missing)	19 (24)
	Mean (SD)	32.89 (25.77)
	Median (min:max)	24.0 (0.0 : 80.0)
	95% CI for mean (t-based)	(20.47, 45.31)
Stage	IB	0 (0.0)
	II	0 (0.0)
	IIA	10 (23.3)
	IIB	33 (76.7)
Surgery type	Whipple	36 (83.7)
	Distal pancreatectomy	6 (14.0)
	Whipple + palliative surgery/bypass	0 (0.0)
	Whipple + other	1 (2.3)
Radiation Reported	No	3 (7.0)
	Yes	40 (93.0)
Other chemotherapy drug	No	4 (9.3)
	Yes	39 (90.7)
Gemcitabine Duration (days)	n (# missing)	43 (0)
	Mean (SD)	97.21 (74.21)
	Median (min:max)	87.0 (7.0 : 344.0)
	95% CI for mean (t-based)	(74.37,120.05)
