# Supplementary material for: Contribution of FKBP5 Genetic Variation to Gemcitabine Treatment and Survival in Pancreatic Adenocarcinoma
Source: PLoS One. 2013 Aug 1;8(8):e70216. doi: 10.1371/journal.pone.0070216 (PMC3731355; doi:10.1371/journal.pone.0070216)
Supplement: Table S2 — (PDF) [file pone.0070216.s005.pdf]

Table S2. List of Next Generation resequenced FKBP5 SNPs. SNPs were compared against databases, such as dbSNP and the "1000 Genomes Project" with "NA" indicating that they were novel to this resequencing study. The gene location described as "Exon 1b","Intron 1b" and "Intron 2b" refers to a longer transcript of FKBP5, BC042605; other gene locations refer to transcript NM_004117.2. Transcription Factor Binding Site: score 0-1000 indicated the signal strength observed in any cell line based on the ENCODE ChIP-seq (chromatin immunoprecipitation with antibodies specific to the transcription factor followed by sequencing of the precipitated DNA) (NCBI36/hg18). Abbreviations: MAF, Minor Allele Frequency; SNP, single nucleotide polymorphism.

SNPs in databases	MAF	Genomic position; hg18	Genomic position; hg19	rs ID	Gene location	Transcription Factor Binding Site
dbSNP/1000 Gen.	0.406	35804864	35696886	rs2766537,rs181508114	5'FR	Score=288;Ini1
1000 Genomes	0.031	35804704	35696726	rs143229535	5'FR	Score=555;c-Myc
dbSNP/1000 Gen.	0.313	35804341	35696363	rs2817035	5'FR	Score=1000;GR,HEY1
dbSNP/1000 Gen.	<0.01	35804267	35696289	rs2817033,rs2817034	Exon 1b	Score=1000;TCF12,GR,HEY1
dbSNP/1000 Gen.	0.406	35804257	35696279	rs2817033,rs2817034	Intron 1b	Score=1000;TCF12,GR,HEY1
dbSNP/1000 Gen.	0.125	35803569	35695591	rs28435135	Intron 1b	Score=1000;TCF12,GR,HEY1,USF-1
NA	<0.01	35803548	35695570	NA	Intron 1b	Score=1000;GR,HEY1,USF-1
dbSNP/1000 Gen.	0.313	35803519	35695541	rs2766536	Intron 1b	Score=1000;GR,HEY1,USF-1
dbSNP/1000 Gen.	0.031	35803252	35695274	rs7751693	Intron 1b	Score=1000;GR,HEY1,USF-1
dbSNP/1000 Gen.	<0.01	35803171	35695193	rs75552415,rs192417627	Intron 1b	Score=1000;GR,HEY1,USF-1
dbSNP/1000 Gen.	0.250	35802904	35694926	rs10947565,rs140967114	Intron 1b	Score=1000;GR
dbSNP/1000 Gen.	0.031	35802555	35694577	rs77677939	Intron 1b	Score=1000;GR
dbSNP/1000 Gen.	0.063	35802223	35694245	rs76917354,rs191346255	Intron 1b	Score=1000;GR
dbSNP/1000 Gen.	<0.01	35802128	35694150	rs115927448	Intron 1b	Score=1000;GR
dbSNP/1000 Gen.	0.250	35800906	35692928	rs12203716	Intron 1b	NA
1000 Genomes	<0.01	35800163	35692185	rs191629931	Intron 1b	NA
dbSNP/1000 Gen.	0.438	35799760	35691782	rs2766535	Intron 1b	NA
dbSNP/1000 Gen.	0.281	35799618	35691640	rs4236047	Intron 1b	NA
dbSNP/1000 Gen.	0.031	35799414	35691436	rs114304008	Intron 1b	NA
dbSNP/1000 Gen.	0.031	35799311	35691333	rs78220087	Intron 1b	NA
1000 Genomes	<0.01	35798367	35690389	rs148378472	Intron 1b	Score=1000;GR
dbSNP/1000 Gen.	0.250	35797848	35689870	rs112633560,rs145112268,rs56362135	Intron 1b	NA
dbSNP/1000 Gen.	0.188	35797810	35689832	rs13198515	Intron 1b	NA
dbSNP/1000 Gen.	0.250	35797771	35689793	rs114531119,rs12206670	Intron 1b	NA
dbSNP/1000 Gen.	0.031	35797716	35689738	rs77168471	Intron 1b	NA
dbSNP/1000 Gen.	0.031	35797371	35689393	rs7747780	Intron 1b	NA
NA	0.469	35797219	35689241	NA	Intron 1b	NA
1000 Genomes	0.344	35797103	35689125	rs11372409	Intron 1b	NA
1000 Genomes	0.250	35796661	35688683	rs144748999,rs144356848,rs10710071	Intron 1b	NA
dbSNP/1000 Gen.	0.281	35796597	35688619	rs2817032	Intron 1b	Score=246;NFKB
1000 Genomes	<0.01	35796280	35688302	rs182725566	Intron 1b	Score=570;Ini1
1000 Genomes	0.031	35795936	35687958	rs112209535,rs113902616	Intron 1b	Score=1000;PU.1
dbSNP/1000 Gen.	0.031	35795915	35687937	rs77379145	Intron 2b	Score=1000;PU.1
1000 Genomes	<0.01	35795815	35687837	rs183347446,rs35951655	Intron 2b	Score=1000;PU.1
dbSNP/1000 Gen.	0.313	35795227	35687249	rs9348981	Intron 2b	Score=322;EBF
dbSNP/1000 Gen.	<0.01	35794922	35686944	rs73729770,rs34581862	Intron 2b	Score=246;NFKB
1000 Genomes	0.031	35794718	35686740	rs75941623	Intron 2b	Score=282;TCF12
dbSNP/1000 Gen.	0.031	35794415	35686437	rs6914582	Intron 2b	Score=450;PU.1

dbSNP/1000 Gen.	0.031	35794369	35686391	rs6914554	Intron 2b	Score=450;PU.1
1000 Genomes	0.031	35794152	35686174	rs143262325	Intron 2b	Score=450;PU.1
dbSNP/1000 Gen.	0.250	35793933	35685955	rs12200498	Intron 2b	Score=450;PU.1
dbSNP/1000 Gen.	0.188	35793692	35685714	rs2766534	Intron 2b	NA
dbSNP/1000 Gen.	0.500	35793468	35685490	rs2766533	Intron 2b	NA
dbSNP/1000 Gen.	0.250	35793171	35685193	rs2817031,rs147048411	Intron 2b	Score=534;Max
dbSNP/1000 Gen.	0.250	35792818	35684840	rs2766532	Intron 2b	NA
1000 Genomes	0.281	35792527	35684549	rs34618058	Intron 2b	NA
dbSNP/1000 Gen.	0.313	35791526	35683548	rs4711429	Intron 2b	Score=1000;GR
dbSNP/1000 Gen.	0.313	35791037	35683059	rs9394314	Intron 2b	NA
dbSNP/1000 Gen.	<0.01	35789861	35681883	rs73729766,rs186745286	Intron 2b	NA
dbSNP/1000 Gen.	0.313	35789755	35681777	rs4713921	Intron 2b	NA
dbSNP/1000 Gen.	<0.01	35789706	35681728	rs57599664	Intron 2b	NA
dbSNP/1000 Gen.	0.313	35788084	35680106	rs6909804	Intron 2b	NA
NA	<0.01	35787976	35679998	NA	Intron 2b	NA
1000 Genomes	0.031	35787670	35679692	rs143018638	Intron 2b	Score=320;CEBPB
NA	0.031	35787509	35679531	NA	Intron 2b	NA
dbSNP/1000 Gen.	0.438	35786601	35678623	rs4713920,rs4711428,rs74370322	Intron 2b	NA
dbSNP/1000 Gen.	0.313	35786597	35678619	rs4713920,rs4711428,rs74370322	Intron 2b	NA
1000 Genomes	0.031	35786126	35678148	rs144583297	Intron 2b	Score=438;c-Fos
dbSNP/1000 Gen.	<0.01	35786032	35678054	rs187009689,rs58580399	Intron 2b	Score=438;c-Fos
dbSNP/1000 Gen.	<0.01	35785763	35677785	rs77644320,rs114807361	Intron 2b	Score=137;STAT1
1000 Genomes	<0.01	35785508	35677530	rs184680207	Intron 2b	NA
dbSNP/1000 Gen.	0.313	35785031	35677053	rs4711427	Intron 2b	NA
dbSNP/1000 Gen.	0.313	35785005	35677027	rs184957353,rs4711426	Intron 2b	NA
dbSNP/1000 Gen.	0.313	35784969	35676991	rs4713919,rs192178086	Intron 2b	NA
dbSNP/1000 Gen.	0.313	35784863	35676885	rs4713918	Intron 2b	NA
dbSNP/1000 Gen.	0.469	35784332	35676354	rs34417388,rs74632259,rs71002596	Intron 2b	NA
dbSNP/1000 Gen.	0.313	35784296	35676318	rs6457842,rs182985165	Intron 2b	NA
dbSNP/1000 Gen.	0.313	35783851	35675873	rs6905674	Intron 2b	NA
dbSNP/1000 Gen.	0.375	35783674	35675696	rs9380529	Intron 2b	NA
dbSNP/1000 Gen.	0.344	35783639	35675661	rs6900592	Intron 2b	NA
1000 Genomes	<0.01	35783584	35675606	rs149588390	Intron 2b	NA
dbSNP/1000 Gen.	0.250	35783557	35675579	rs55694295	Intron 2b	NA
1000 Genomes	<0.01	35783272	35675294	rs186264450,rs144864436	Intron 2b	NA
NA	<0.01	35783168	35675190	NA	Intron 2b	NA
dbSNP/1000 Gen.	0.344	35783059	35675081	rs185998144,rs9296160	Intron 2b	NA
dbSNP/1000 Gen.	0.375	35783013	35675035	rs9470084	Intron 2b	NA
dbSNP/1000 Gen.	0.344	35782823	35674845	rs9462104	Intron 2b	NA
dbSNP/1000 Gen.	0.313	35782595	35674617	rs9394313	Intron 2b	Score=701;c-Jun
dbSNP/1000 Gen.	0.469	35781310	35673332	rs13213010	Intron 2b	NA
1000 Genomes	<0.01	35780890	35672912	rs184569594	Intron 2b	NA
1000 Genomes	0.031	35780536	35672558	rs192409932	Intron 2b	NA
dbSNP/1000 Gen.	0.375	35780465	35672487	rs34727090,rs58212271,rs77399846	Intron 2b	NA
1000 Genomes	<0.01	35780435	35672457	rs187639919	Intron 2b	NA
dbSNP/1000 Gen.	0.469	35780348	35672370	s142075046,rs35718174,rs77043892,rs5780813	Intron 2b	NA

dbSNP/1000 Gen.	0.469	35780308	35672330	rs9394312	Intron 2b	NA
NA	<0.01	35779935	35671957	NA	Intron 2b	NA
1000 Genomes	<0.01	35779689	35671711	rs76669992,rs191917844,rs184760557	Intron 2b	NA
dbSNP/1000 Gen.	0.250	35779629	35671651	rs10456432	Intron 2b	Score=1000;USF-1
dbSNP/1000 Gen.	0.375	35779143	35671165	rs2395635	Intron 2b	NA
dbSNP/1000 Gen.	0.438	35778999	35671021	rs7745324	Intron 2b	NA
dbSNP/1000 Gen.	0.469	35778585	35670607	rs6902321	Intron 2b	NA
dbSNP/1000 Gen.	0.250	35778454	35670476	rs12190582	Intron 2b	NA
NA	<0.01	35778443	35670465	NA	Intron 2b	NA
1000 Genomes	0.250	35778129	35670151	49392522,rs35311317,rs148861980,rs1124377[illegible]	Intron 2b	NA
1000 Genomes	0.063	35778124	35670146	[illegible]64,rs149392522,rs35311317,rs148861980,rs112[illegible]	Intron 2b	NA
dbSNP/1000 Gen.	0.375	35777961	35669983	rs4713916	Intron 2b	NA
NA	0.031	35777852	35669874	NA	Intron 2b	NA
1000 Genomes	0.031	35777525	35669547	rs144827927	Intron 2b	NA
dbSNP/1000 Gen.	0.406	35777288	35669310	[illegible]s9470082,rs4713915,rs184867022,rs18995787[illegible]	Intron 2b	NA
dbSNP/1000 Gen.	0.031	35777278	35669300	rs9470082,rs4713915	Intron 2b	NA
dbSNP/1000 Gen.	0.406	35777122	35669144	rs4713914,rs75172019	Intron 2b	NA
dbSNP/1000 Gen.	0.406	35776563	35668585	rs9394311	Intron 2b	NA
1000 Genomes	<0.01	35776478	35668500	rs186976823,rs140078496,rs143691075	Intron 2b	NA
1000 Genomes	<0.01	35776462	35668484	rs183344228	Intron 2b	NA
1000 Genomes	0.031	35776273	35668295	rs183557661,rs145987281	Intron 2b	NA
1000 Genomes	0.031	35776119	35668141	rs115646474	Intron 2b	NA
dbSNP/1000 Gen.	0.375	35775969	35667991	rs12153967	Intron 2b	NA
dbSNP/1000 Gen.	0.375	35775838	35667860	rs943297	Intron 2b	NA
dbSNP/1000 Gen.	0.031	35774523	35666545	rs77319078	Intron 2b	NA
dbSNP/1000 Gen.	0.031	35773746	35665768	rs13340463	Intron 2b	NA
dbSNP/1000 Gen.	0.438	35773174	35665196	rs4713911	Intron 2b	NA
dbSNP/1000 Gen.	0.031	35772983	35665005	rs80179277	Intron 2b	NA
1000 Genomes	<0.01	35772531	35664553	rs147130038	Intron 2b	NA
dbSNP/1000 Gen.	0.344	35772230	35664252	rs137986902,rs9380528	Intron 2b	NA
NA	<0.01	35772111	35664133	NA	Intron 2b	NA
NA	0.031	35771396	35663418	NA	Intron 2b	NA
1000 Genomes	0.031	35771004	35663026	rs184981757,rs150456373	Intron 2b	NA
dbSNP/1000 Gen.	0.375	35770631	35662653	rs56311918	Intron 2b	Score=1000;JunD
dbSNP/1000 Gen.	0.375	35770084	35662106	rs7763535	Intron 2b	NA
dbSNP/1000 Gen.	0.250	35770010	35662032	rs55987213	Intron 2b	NA
1000 Genomes	0.031	35769956	35661978	rs146257681	Intron 2b	NA
dbSNP/1000 Gen.	0.375	35769797	35661819	rs7759392	Intron 2b	NA
dbSNP/1000 Gen.	0.219	35769411	35661433	rs35253763,rs144395988,rs114191749	Intron 2b	NA
NA	0.031	35769207	35661229	NA	Intron 2b	NA
1000 Genomes	0.063	35768679	35660701	rs147225648,rs140576483	Intron 2b	NA
1000 Genomes	<0.01	35768574	35660596	rs71002588,rs187208328	Intron 2b	NA
dbSNP/1000 Gen.	0.031	35767825	35659847	rs78695908,rs62402145	Intron 2b	NA
1000 Genomes	<0.01	35767224	35659246	rs141203278	Intron 2b	Score=80;USF-1
dbSNP/1000 Gen.	0.375	35766622	35658644	rs9368885,rs138974591	Intron 2b	NA
1000 Genomes	0.031	35766403	35658425	rs141436620,rs185669307	Intron 2b	NA

dbSNP/1000 Gen.	0.375	35766305	35658327	rs9380526	Intron 2b	NA
dbSNP/1000 Gen.	0.375	35764541	35656563	rs9462103	UTR5	Score=1000;SP1,HEY1,TAF1,POU2F2
dbSNP/1000 Gen.	0.219	35764409	35656431	rs13215797	Intron 1	Score=1000;HEY1,TAF1,POU2F2
NA	<0.01	35764231	35656253	NA	Intron 1	Score=1000;HEY1,BAF170,TAF1
1000 Genomes	<0.01	35763962	35655984	rs10947564,rs111406566	Intron 1	Score=1000;BAF170,TAF1
dbSNP/1000 Gen.	0.094	35763952	35655974	rs10947564	Intron 1	Score=1000;BAF170,TAF1
dbSNP/1000 Gen.	0.375	35763223	35655245	rs3800372	Intron 1	Score=594;PU.1
dbSNP/1000 Gen.	0.375	35761415	35653437	rs189114608,rs146123716,rs10947563	Intron 1	NA
dbSNP/1000 Gen.	<0.01	35760898	35652920	rs115634614	Intron 1	NA
1000 Genomes	<0.01	35760871	35652893	rs77444496,rs187624235	Intron 1	NA
dbSNP/1000 Gen.	<0.01	35760821	35652843	rs74432203,rs34110646	Intron 1	NA
dbSNP/1000 Gen.	0.344	35760816	35652838	rs74432203,rs34110646	Intron 1	NA
dbSNP/1000 Gen.	0.438	35759965	35651987	rs6899478	Intron 1	NA
1000 Genomes	0.031	35759261	35651283	rs147614773	Intron 1	NA
dbSNP/1000 Gen.	0.031	35759185	35651207	rs76019376	Intron 1	NA
1000 Genomes	0.031	35758265	35650287	rs142232122,rs151229770	Intron 1	NA
1000 Genomes	0.031	35758047	35650069	rs34218355	Intron 1	NA
dbSNP/1000 Gen.	0.375	35756808	35648830	rs6457839	Intron 1	NA
1000 Genomes	0.031	35756071	35648093	rs142989415	Intron 1	NA
1000 Genomes	<0.01	35755572	35647594	rs185290009	Intron 1	NA
dbSNP/1000 Gen.	0.031	35755288	35647310	rs4713908	Intron 1	NA
dbSNP/1000 Gen.	0.406	35754413	35646435	rs9470080,rs183332973	Intron 1	NA
dbSNP/1000 Gen.	0.406	35753063	35645085	rs7758906	Intron 1	NA
dbSNP/1000 Gen.	0.031	35751701	35643723	rs73748221	Intron 1	NA
dbSNP/1000 Gen.	0.031	35751398	35643420	rs6931036,rs73748220	Intron 1	NA
dbSNP/1000 Gen.	0.031	35751395	35643417	rs6931036,rs73748220	Intron 1	NA
1000 Genomes	<0.01	35751293	35643315	rs141473268	Intron 1	NA
1000 Genomes	0.031	35751239	35643261	rs148234137	Intron 1	NA
dbSNP/1000 Gen.	0.031	35751053	35643075	rs4713907	Intron 1	NA
dbSNP/1000 Gen.	0.094	35751041	35643063	rs9470079	Intron 1	NA
dbSNP/1000 Gen.	<0.01	35749202	35641224	rs75477235	Intron 1	NA
1000 Genomes	<0.01	35748720	35640742	rs185571730,rs190226916,rs143224792	Intron 1	NA
1000 Genomes	0.188	35748516	35640538	[illegible]135,rs11399067,rs71830530,rs139485621,rs14[illegible]	Intron 1	NA
NA	0.031	35748247	35640269	NA	Intron 1	NA
1000 Genomes	<0.01	35747785	35639807	rs142423206	Intron 1	NA
1000 Genomes	0.031	35747671	35639693	rs9394310,rs182596274	Intron 1	NA
dbSNP/1000 Gen.	0.438	35747666	35639688	rs9394310,rs182596274	Intron 1	NA
dbSNP/1000 Gen.	0.031	35747387	35639409	rs184663706	Intron 1	NA
dbSNP/1000 Gen.	0.313	35746954	35638976	rs9368882	Intron 1	NA
dbSNP/1000 Gen.	0.031	35743496	35635518	rs7752084	Intron 1	NA
dbSNP/1000 Gen.	<0.01	35742835	35634857	rs189097850	Intron 1	NA
1000 Genomes	<0.01	35742452	35634474	rs76065176	Intron 1	NA
dbSNP/1000 Gen.	0.406	35742266	35634288	rs9368881	Intron 1	NA
dbSNP/1000 Gen.	<0.01	35741434	35633456	rs13192954	Intron 1	Score=58;NFKB
dbSNP/1000 Gen.	0.406	35741016	35633038	rs9380525	Intron 1	NA
dbSNP/1000 Gen.	<0.01	35740895	35632917	rs75710780	Intron 1	Score=72;NFKB

1000 Genomes	0.125	35740361	35632383	rs137977904	Intron 1	NA
1000 Genomes	0.031	35739970	35631992	rs34853875	Intron 1	NA
dbSNP/1000 Gen.	0.063	35737387	35629409	rs111816824,rs111348833,rs112972898	Intron 1	NA
dbSNP/1000 Gen.	0.063	35737296	35629318	rs111927248	Intron 1	NA
dbSNP/1000 Gen.	0.063	35737178	35629200	rs7747647	Intron 1	NA
1000 Genomes	<0.01	35736502	35628524	rs151038510,rs111367541	Intron 1	NA
dbSNP/1000 Gen.	0.063	35736456	35628478	rs4713905,rs188123934	Intron 1	NA
1000 Genomes	0.031	35735759	35627781	rs148451847	Intron 1	NA
dbSNP/1000 Gen.	0.063	35735245	35627267	rs7775489	Intron 1	NA
dbSNP/1000 Gen.	0.063	35734910	35626932	rs77253887	Intron 1	NA
dbSNP/1000 Gen.	0.063	35734478	35626500	rs4711425	Intron 1	NA
dbSNP/1000 Gen.	0.219	35733914	35625936	rs13215497	Intron 1	NA
dbSNP/1000 Gen.	0.406	35733683	35625705	rs6929523	Intron 1	NA
dbSNP/1000 Gen.	<0.01	35733616	35625638	rs79476859	Intron 1	NA
dbSNP/1000 Gen.	0.406	35733125	35625147	rs4713904	Intron 1	NA
dbSNP/1000 Gen.	0.469	35732811	35624833	rs35090133,rs71002584	Intron 1	NA
dbSNP/1000 Gen.	0.250	35732689	35624711	rs13207605,rs12197246	Intron 1	NA
1000 Genomes	0.125	35732606	35624628	rs141720418,rs191631236	Intron 1	NA
dbSNP/1000 Gen.	0.438	35732521	35624543	rs9296159	Intron 1	NA
NA	<0.01	35732325	35624347	NA	Intron 1	NA
NA	<0.01	35730418	35622440	NA	Intron 1	NA
dbSNP/1000 Gen.	0.031	35730185	35622207	rs2092427	Intron 1	NA
dbSNP/1000 Gen.	0.063	35729899	35621921	rs17614642	Intron 1	NA
dbSNP/1000 Gen.	0.375	35729759	35621781	rs9394309	Intron 1	NA
1000 Genomes	0.031	35728735	35620757	rs183216045	Intron 1	NA
1000 Genomes	<0.01	35728734	35620756	rs183216045	Intron 1	NA
dbSNP/1000 Gen.	0.250	35728605	35620627	rs10456431	Intron 1	NA
dbSNP/1000 Gen.	0.219	35728550	35620572	rs11754441	Intron 1	NA
dbSNP/1000 Gen.	0.406	35728528	35620550	rs6931118	Intron 1	NA
NA	<0.01	35728152	35620174	NA	Intron 1	NA
dbSNP/1000 Gen.	0.375	35727956	35619978	rs4544902	Intron 1	NA
dbSNP/1000 Gen.	0.031	35727532	35619554	rs1475774	Intron 1	Score=474;Brg1
dbSNP/1000 Gen.	<0.01	35726664	35618686	rs77571794	Intron 1	Score=474;Brg1
1000 Genomes	0.063	35726280	35618302	rs140253283	Intron 1	NA
dbSNP/1000 Gen.	0.375	35725799	35617821	rs185661038,rs4713903	Intron 1	NA
dbSNP/1000 Gen.	0.375	35725563	35617585	rs6912833	Intron 1	NA
dbSNP/1000 Gen.	0.406	35725188	35617210	478,rs11414221,rs71725261,rs143661600,rs13	Intron 1	NA
1000 Genomes	<0.01	35724864	35616886	rs113425178,rs191016418	Intron 1	NA
dbSNP/1000 Gen.	0.406	35724644	35616666	rs9357201,rs9357202	Intron 1	NA
dbSNP/1000 Gen.	0.063	35723845	35615867	rs66503860,rs57126691	Intron 1	NA
dbSNP/1000 Gen.	0.031	35723690	35615712	rs181472746,rs9462100	Intron 1	NA
dbSNP/1000 Gen.	0.063	35723108	35615130	rs1334894	Intron 1	Score=103;PU.1
1000 Genomes	<0.01	35722862	35614884	rs188632512	Intron 1	NA
dbSNP/1000 Gen.	0.250	35722722	35614744	rs17542466	Intron 1	NA
1000 Genomes	0.063	35722504	35614526	rs140656232	Intron 1	NA
dbSNP/1000 Gen.	0.031	35722306	35614328	rs187521165,rs59595954	Intron 1	NA

dbSNP/1000 Gen.	0.031	35722105	35614127	rs73748211	Intron 1	NA
dbSNP/1000 Gen.	0.219	35722004	35614026	rs4713902	Intron 1	NA
dbSNP/1000 Gen.	0.031	35721976	35613998	rs7771722	Intron 1	NA
dbSNP/1000 Gen.	0.031	35721310	35613332	rs73748209	Intron 1	NA
dbSNP/1000 Gen.	0.031	35721176	35613198	rs9767565	Intron 1	NA
1000 Genomes	<0.01	35720889	35612911	rs148128369	Intron 1	Score=385;NFKB
NA	0.031	35719458	35611480	NA	Intron 1	NA
dbSNP/1000 Gen.	0.250	35719210	35611232	rs9394307,rs58549426	Intron 1	NA
dbSNP/1000 Gen.	0.063	35718729	35610751	rs12527329	Intron 1	NA
dbSNP/1000 Gen.	0.344	35718659	35610681	rs2143404,rs35448780	Intron 1	NA
1000 Genomes	0.031	35718531	35610553	rs148043129	Exon 2	NA
dbSNP/1000 Gen.	0.031	35718318	35610340	rs12110366	Intron 2	NA
dbSNP/1000 Gen.	0.438	35718286	35610308	rs6902124,rs111511988	Intron 2	NA
dbSNP/1000 Gen.	0.031	35718242	35610264	rs187825034,rs113065020	Intron 2	NA
dbSNP/1000 Gen.	0.219	35718193	35610215	rs9348979,rs28558535	Intron 2	NA
dbSNP/1000 Gen.	0.063	35717792	35609814	rs7756437,rs142708384	Intron 2	NA
1000 Genomes	0.031	35717519	35609541	NA	Intron 2	NA
1000 Genomes	0.031	35716324	35608346	NA	Intron 2	NA
1000 Genomes	<0.01	35716217	35608239	rs140140532	Intron 2	Score=1000;GR
dbSNP/1000 Gen.	0.063	35716074	35608096	rs55922240	Intron 2	Score=1000;GR
1000 Genomes	0.031	35715938	35607960	rs73748206,rs186158240	Intron 2	Score=1000;GR
dbSNP/1000 Gen.	0.031	35715933	35607955	rs73748206	Intron 2	Score=1000;GR
dbSNP/1000 Gen.	0.031	35715599	35607621	rs7763114	Intron 2	NA
dbSNP/1000 Gen.	0.406	35715549	35607571	rs1360780	Intron 2	NA
1000 Genomes	0.031	35715307	35607329	rs115214997,rs188105805	Intron 2	NA
dbSNP/1000 Gen.	0.031	35714379	35606401	rs58873316	Intron 2	NA
1000 Genomes	0.031	35714013	35606035	rs151140827	Intron 2	NA
dbSNP/1000 Gen.	0.406	35713178	35605200	rs7751598	Intron 2	NA
dbSNP/1000 Gen.	0.031	35712673	35604695	rs73748205	Intron 3	NA
dbSNP/1000 Gen.	0.313	35712623	35604645	rs7746850	Intron 3	NA
dbSNP/1000 Gen.	0.406	35712085	35604107	rs1591365	Intron 3	NA
dbSNP/1000 Gen.	0.031	35711567	35603589	rs72921237	Intron 3	NA
dbSNP/1000 Gen.	0.313	35711097	35603119	rs7760951	Intron 3	NA
dbSNP/1000 Gen.	<0.01	35710973	35602995	rs112754019	Intron 3	NA
dbSNP/1000 Gen.	0.344	35710950	35602972	rs7740395	Intron 3	NA
dbSNP/1000 Gen.	0.406	35710471	35602493	[illegible]s66500202,rs79702774,rs79193426,rs6690757[illegible]	Intron 3	NA
1000 Genomes	<0.01	35709774	35601796	rs182564277	Intron 3	NA
dbSNP/1000 Gen.	0.406	35709754	35601776	rs3798347	Intron 3	NA
dbSNP/1000 Gen.	0.031	35709507	35601529	rs28675670	Intron 3	NA
NA	0.031	35707732	35599754	NA	Intron 3	NA
dbSNP/1000 Gen.	0.031	35705868	35597890	rs74636231	Intron 3	NA
dbSNP/1000 Gen.	0.031	35705681	35597703	rs16879378	Intron 3	NA
dbSNP/1000 Gen.	0.063	35704890	35596912	rs10947562	Intron 3	NA
dbSNP/1000 Gen.	0.031	35703459	35595481	rs141064452	Intron 3	NA
dbSNP/1000 Gen.	0.063	35702819	35594841	rs181392730,rs76212347	Intron 3	NA
1000 Genomes	0.031	35702644	35594666	rs144714430	Intron 3	NA

dbSNP/1000 Gen.	0.031	35701961	35593983	rs7747121	Intron 3	NA
NA	<0.01	35701873	35593895	NA	Intron 3	NA
dbSNP/1000 Gen.	0.031	35701836	35593858	rs7743425	Intron 3	NA
1000 Genomes	<0.01	35700795	35592817	rs190824366	Intron 3	NA
dbSNP/1000 Gen.	0.313	35700722	35592744	rs7748266	Intron 3	NA
dbSNP/1000 Gen.	<0.01	35700518	35592540	rs79134986	Intron 3	NA
dbSNP/1000 Gen.	0.375	35700253	35592275	1025,rs35508106,rs139922231,rs60605988,rs34	Intron 3	Score=173;CTCF
dbSNP/1000 Gen.	0.063	35699311	35591333	rs62402121	Intron 3	NA
dbSNP/1000 Gen.	0.469	35698890	35590912	rs184674431,rs4713900	Intron 3	NA
dbSNP/1000 Gen.	0.031	35698369	35590391	rs16879318,rs111355550	Intron 3	NA
dbSNP/1000 Gen.	0.031	35698253	35590275	rs113805447	Intron 3	NA
dbSNP/1000 Gen.	0.031	35698135	35590157	rs75118012,rs151248491	Intron 3	NA
dbSNP/1000 Gen.	0.063	35698070	35590092	rs7754668	Intron 3	NA
dbSNP/1000 Gen.	0.063	35697615	35589637	rs72921231	Intron 3	NA
dbSNP/1000 Gen.	0.063	35697048	35589070	rs9380524	Intron 3	NA
dbSNP/1000 Gen.	0.031	35696209	35588231	rs74682753	Intron 3	NA
NA	<0.01	35694697	35586719	NA	Intron 4	NA
dbSNP/1000 Gen.	0.063	35694351	35586373	rs747411	Intron 5	NA
dbSNP/1000 Gen.	0.406	35693592	35585614	rs9368878	Intron 5	NA
dbSNP/1000 Gen.	0.031	35693257	35585279	rs79285437	Intron 5	NA
dbSNP/1000 Gen.	0.375	35693077	35585099	rs12527893,rs72065011	Intron 5	NA
dbSNP/1000 Gen.	0.031	35692412	35584434	rs73748204	Intron 5	NA
dbSNP/1000 Gen.	0.313	35692033	35584055	rs4401662	Intron 5	NA
NA	0.031	35691495	35583517	NA	Intron 5	NA
NA	<0.01	35691301	35583323	NA	Intron 5	NA
1000 Genomes	0.063	35690739	35582761	rs149450199	Intron 5	NA
dbSNP/1000 Gen.	0.063	35690639	35582661	rs9470069	Intron 5	NA
dbSNP/1000 Gen.	0.031	35689864	35581886	rs76813839	Intron 5	NA
dbSNP/1000 Gen.	0.063	35689781	35581803	72913427,rs150466730,rs114317890,rs1912343	Intron 5	NA
dbSNP/1000 Gen.	0.031	35689604	35581626	rs73748203	Intron 5	NA
1000 Genomes	<0.01	35689544	35581566	rs185628664	Intron 5	NA
dbSNP/1000 Gen.	0.031	35688514	35580536	rs190545247,rs75419538	Intron 5	NA
NA	0.031	35688376	35580398	NA	Intron 5	NA
dbSNP/1000 Gen.	0.313	35688276	35580298	rs6457836	Intron 5	NA
dbSNP/1000 Gen.	0.031	35687523	35579545	rs114287508	Intron 5	NA
dbSNP/1000 Gen.	0.313	35687353	35579375	rs6926133	Intron 5	NA
dbSNP/1000 Gen.	0.313	35686980	35579002	rs3777747,rs117730957	Intron 5	NA
dbSNP/1000 Gen.	0.031	35686829	35578851	rs73746499	Intron 5	Score=604;GR
dbSNP/1000 Gen.	<0.01	35686808	35578830	rs116796504	Intron 5	Score=604;GR
dbSNP/1000 Gen.	0.031	35686162	35578184	rs138734514,rs113080387	Intron 5	NA
NA	0.438	35684932	35576954	NA	Intron 5	NA
dbSNP/1000 Gen.	0.031	35684834	35576856	rs145658404,rs9470067	Intron 5	NA
dbSNP/1000 Gen.	<0.01	35684479	35576501	rs142359398	Intron 5	NA
1000 Genomes	0.031	35684023	35576045	rs147348090	Intron 5	NA
dbSNP/1000 Gen.	0.313	35683896	35575918	rs10807152	Intron 5	NA
dbSNP/1000 Gen.	0.063	35683685	35575707	rs72913423	Intron 5	NA

dbSNP/1000 Gen.	0.031	35683634	35575656	rs11966198	Intron 5	NA
dbSNP/1000 Gen.	0.219	35683465	35575487	rs737054	Intron 5	NA
1000 Genomes	0.031	35683016	35575038	rs141686434	Intron 5	NA
1000 Genomes	<0.01	35682638	35574660	rs144360091	Intron 5	NA
NA	0.031	35682615	35574637	NA	Intron 5	NA
dbSNP/1000 Gen.	0.031	35682021	35574043	rs74709645	Intron 5	Score=1000;c-Fos
dbSNP/1000 Gen.	0.031	35681866	35573888	rs114560337	Intron 5	Score=1000;c-Fos
1000 Genomes	0.031	35681172	35573194	rs60069071	Intron 5	NA
dbSNP/1000 Gen.	0.031	35680627	35572649	rs111311633	Intron 5	NA
1000 Genomes	0.031	35680605	35572627	rs149903675	Intron 5	NA
1000 Genomes	0.031	35679933	35571955	rs142034866	Intron 5	NA
dbSNP/1000 Gen.	0.031	35679450	35571472	rs78685770	Intron 5	Score=709;FOSL2
dbSNP/1000 Gen.	0.031	35678997	35571019	rs189185201	Intron 5	Score=709;FOSL2
NA	<0.01	35678715	35570737	NA	Intron 5	NA
dbSNP/1000 Gen.	0.031	35678460	35570482	rs75901890,rs73746498	Intron 5	Score=38;NFKB
dbSNP/1000 Gen.	<0.01	35678384	35570406	rs115061314	Intron 5	Score=149;CEBPB
1000 Genomes	0.031	35677924	35569946	39,rs113292576,rs146184357,rs148544670,rs1[illegible]	Intron 5	Score=1000;GR
dbSNP/1000 Gen.	0.063	35677917	35569939	39,rs113292576,rs146184357,rs148544670,rs1[illegible]	Intron 5	Score=1000;GR
1000 Genomes	0.031	35677564	35569586	rs146067306	Intron 5	Score=1000;GR
dbSNP/1000 Gen.	0.094	35677540	35569562	rs16878812	Intron 5	Score=1000;GR
dbSNP/1000 Gen.	0.313	35677259	35569281	rs183665971,rs4713899	Intron 5	Score=539;STAT1
dbSNP/1000 Gen.	0.031	35677097	35569119	rs149003372,rs16878806	Intron 5	Score=539;STAT1
1000 Genomes	<0.01	35676859	35568881	rs142525835	Intron 5	NA
dbSNP/1000 Gen.	<0.01	35675882	35567904	rs77062724,rs79149296	Intron 5	NA
dbSNP/1000 Gen.	0.406	35675738	35567760	rs2395634,rs149849740	Intron 5	NA
dbSNP/1000 Gen.	0.313	35675642	35567664	rs2395633	Intron 5	NA
NA	0.031	35675513	35567535	NA	Intron 5	NA
dbSNP/1000 Gen.	0.406	35675060	35567082	rs9296158,rs11961905	Intron 5	NA
dbSNP/1000 Gen.	0.375	35674866	35566888	rs66525542,rs57449934	Intron 5	NA
dbSNP/1000 Gen.	0.031	35674126	35566148	rs111499370	Intron 5	NA
dbSNP/1000 Gen.	0.031	35674111	35566133	rs147472748,rs113805226	Intron 5	NA
dbSNP/1000 Gen.	0.031	35673999	35566021	rs76787105	Intron 5	NA
dbSNP/1000 Gen.	0.031	35673715	35565737	rs78596668	Intron 5	NA
1000 Genomes	<0.01	35673697	35565719	rs79208404	Intron 6	NA
dbSNP/1000 Gen.	0.313	35673400	35565422	rs7753746	Intron 6	NA
1000 Genomes	0.031	35673374	35565396	rs150380552	Intron 6	NA
dbSNP/1000 Gen.	0.031	35672403	35564425	rs73746495	Intron 6	Score=877;BAF155
1000 Genomes	0.031	35672238	35564260	rs143268715,rs76042823	Intron 6	Score=877;BAF155
1000 Genomes	<0.01	35671770	35563792	rs148775772	Intron 6	NA
dbSNP/1000 Gen.	0.281	35670952	35562974	rs9366890	Intron 6	NA
dbSNP/1000 Gen.	0.250	35670618	35562640	rs3798346	Intron 6	NA
dbSNP/1000 Gen.	0.313	35670449	35562471	rs3798345	Intron 6	NA
dbSNP/1000 Gen.	<0.01	35669027	35561049	rs77962862	Intron 6	NA
dbSNP/1000 Gen.	0.063	35668619	35560641	rs35236464,rs148477587,rs5875521	Intron 6	NA
1000 Genomes	0.031	35668035	35560057	rs7754690,rs146444873,rs34266330	Intron 6	NA
dbSNP/1000 Gen.	0.031	35668026	35560048	rs7754690,rs146444873	Intron 6	NA

dbSNP/1000 Gen.	0.063	35667751	35559773	rs10498734,rs188212504	Intron 6	NA
1000 Genomes	0.031	35665809	35557831	rs140730875	Intron 7	NA
dbSNP/1000 Gen.	<0.01	35664887	35556909	8051,rs58812576,rs72406704,rs71002575,rs79[illegible]	Intron 7	NA
1000 Genomes	0.031	35664843	35556865	rs36223620	Intron 7	NA
dbSNP/1000 Gen.	0.250	35664498	35556520	rs7771727	Intron 7	NA
NA	0.219	35663769	35555791	NA	Intron 7	NA
dbSNP/1000 Gen.	0.250	35663161	35555183	rs992105	Intron 7	NA
dbSNP/1000 Gen.	0.063	35663088	35555110	rs188553825,rs2294807	Intron 7	NA
dbSNP/1000 Gen.	0.031	35663034	35555056	rs186372061,rs73746494	Intron 7	NA
dbSNP/1000 Gen.	0.031	35662244	35554266	rs77612799	Intron 8	NA
NA	0.063	35661339	35553361	NA	Intron 8	NA
dbSNP/1000 Gen.	0.031	35661323	35553345	rs144537644,rs73746492,rs73746493	Intron 8	NA
dbSNP/1000 Gen.	0.031	35661322	35553344	rs144537644,rs73746492,rs73746493	Intron 8	NA
dbSNP/1000 Gen.	0.031	35660605	35552627	rs16878591	Intron 8	NA
dbSNP/1000 Gen.	0.031	35660167	35552189	rs73746491	Intron 8	NA
dbSNP/1000 Gen.	0.031	35659391	35551413	rs7755289	Intron 8	NA
dbSNP/1000 Gen.	0.031	35658893	35550915	rs59320339	Intron 8	NA
1000 Genomes	0.031	35658834	35550856	rs10631894,rs34717528	Intron 8	NA
dbSNP/1000 Gen.	0.031	35658181	35550203	rs73746490	Intron 8	NA
dbSNP/1000 Gen.	0.031	35657920	35549942	rs112124918	Intron 8	NA
dbSNP/1000 Gen.	0.063	35657648	35549670	rs115829819,rs755658,rs184828383	Intron 8	NA
1000 Genomes	<0.01	35657602	35549624	rs140458482	Intron 8	NA
dbSNP/1000 Gen.	0.031	35656386	35548408	rs111674784	Intron 8	NA
dbSNP/1000 Gen.	0.375	35656214	35548236	rs7757037	Intron 8	NA
dbSNP/1000 Gen.	<0.01	35655201	35547223	rs146846979,rs114356859	Intron 9	NA
dbSNP/1000 Gen.	0.031	35654872	35546894	rs61188051	Intron 9	Score=67;GR
dbSNP/1000 Gen.	0.063	35654844	35546866	rs72913417	Intron 9	Score=67;GR
1000 Genomes	0.250	35654729	35546751	[illegible]132,rs67988585,rs149689108,rs10626273,rs34	Intron 9	Score=67;GR
dbSNP/1000 Gen.	0.031	35654383	35546405	rs111375581	Intron 9	NA
NA	<0.01	35654052	35546074	NA	Intron 9	NA
1000 Genomes	<0.01	35653979	35546001	rs188508079	Intron 9	NA
NA	0.031	35653682	35545704	NA	Intron 9	NA
dbSNP/1000 Gen.	0.031	35652920	35544942	rs143893856,rs34866878	Exon 10	NA
dbSNP/1000 Gen.	0.031	35652867	35544889	rs146787424	Exon 10	NA
dbSNP/1000 Gen.	0.031	35652008	35544030	rs56002954	Intron 10	NA
dbSNP/1000 Gen.	0.031	35651973	35543995	rs45586932	Intron 10	NA
dbSNP/1000 Gen.	0.406	35651198	35543220	rs111803919,rs146420608	3'UTR	NA
dbSNP/1000 Gen.	0.063	35650504	35542526	rs11545925	3'UTR	NA
dbSNP/1000 Gen.	0.344	35650454	35542476	rs3800373	3'UTR	NA
dbSNP/1000 Gen.	0.031	35650023	35542045	rs41270080	3'UTR	NA
dbSNP/1000 Gen.	0.188	35649410	35541432	[illegible]s188433627,rs66957944,rs1043805,rs1154592[illegible]	3'UTR	NA
NA	<0.01	35649098	35541120	NA	3'FR	NA
dbSNP/1000 Gen.	0.344	35649036	35541058	rs12055438	3'FR	NA
dbSNP/1000 Gen.	0.250	35648846	35540868	rs9470063	3'FR	NA
