# Supplementary material for: Contribution of FKBP5 Genetic Variation to Gemcitabine Treatment and Survival in Pancreatic Adenocarcinoma
Source: PLoS One. 2013 Aug 1;8(8):e70216. doi: 10.1371/journal.pone.0070216 (PMC3731355; doi:10.1371/journal.pone.0070216)
Supplement: Table S3 — (PDF) [file pone.0070216.s006.pdf]

Table S3. Human FKBP5 gene Sanger resequencing. Polymorphisms in exons (2-11) and untranslated regions (UTRs) are numbered relative to the “A” nucleotide in the translation initiation codon in exon 2. Variant nucleotides located within introns are numbered based on their distance from the nearest splice junction, with negative numbers assigned to positions 5’ to that location, and positive numbers for positions 3’.

FKBP5 Polymorphisms

				Frequency of Minor Allele	
**Location**	**Nucleotide**	**Sequence Change**	**Amino Acid Change**	**Pancreatic Samples**	**RefSNP ID**
5'FR	-40325	A→G		0.444	rs2766537
5'FR	-40165	G→A		0.016	
5'FR	-39802	C→T		0.315	rs2817035
5'FR	-39728	A→G			rs2817034
5'FR	-39718	G→A		0.444	rs2817033
5'FR	-31741	C→A		0.008	
5'FR	-31397	C→T		0.016	
Intron 1	18	G→A		0.347	rs9462103
Intron 1	150	G→A		0.145	rs13215797
Intron 1	478	C→G		0.008	
Intron 1	597	C→G		0.008	
Intron 1	607	A→G		0.194	rs10947564
Intron 1	-131	T→A		0.056	rs12527329
Intron 1	-61	G→A		0.258	rs2143404
Exon 2	49	A→G	Thr(17)Ala	0.016	
Intron 2	157	G→A		0.040	rs12110366
Intron 2	189	T→G		0.371	rs6902124
Intron 3	96	A→T		0.040	rs73748205
Intron 3	146	C→T		0.266	rs7746850
Exon 10	1095	C→T		0.040	rs34866878
Exon 10	1148	A→T	Gln(383)Leu	0.016	
Intron 10	-311	A→C		0.040	rs56002954
Intron 10	-276	C→T		0.040	rs45586932
