# Supplementary material for: Contribution of FKBP5 Genetic Variation to Gemcitabine Treatment and Survival in Pancreatic Adenocarcinoma
Source: PLoS One. 2013 Aug 1;8(8):e70216. doi: 10.1371/journal.pone.0070216 (PMC3731355; doi:10.1371/journal.pone.0070216)
Supplement: Table S4 — (PDF) [file pone.0070216.s007.pdf]

Table S4. Oligonucleotide sequences for site-directed mutagenesis and reporter gene assay, and EMSA probes. (A) Site-directed mutagenesis oligonucleotide sequences. Underlined nucleotides are responsible for amino acid changes. (B) Sequences of oligonucleotides used to perform PCR reactions to amplify regions surrounding indicated SNPs for luciferase reporter gene constructs using patients DNA as a template. Underlined nucleotides represent the restriction enzyme site, Acc65I for forward primer and MIu1 for reverse. (C) EMSA probes.

A) Site Directed Mutagenesis Primers

SNP (FKBP5)		*Probe Sequence*
Thr(22)Ala	forward	5'- GAAAGCCCCACAGCCGCTGTTGCTGAGCAGG-3'
	reverse	5'- CCTGCTCAGCAACAGCGGCTGTGGGGCTTTC-3'
Glu(383)Leu	forward	5'- GGAAGTAAACCCCCTGAATAAGGCTGCAAG-3'
	reverse	5'- CTTGCAGCCTTATTCAGGGGGTTTACTTCC-3'

B) Reporter gene assay

SNP		*Oligonucleotide Sequence*	*product size (bp)*
rs148128369	forward	5'- TAGGTACCTTTCCTGGGGATTCACA-3'	*469*
	reverse	5'- TAACGCGTAGTCCTTCCATCTCA-3'	
rs116796504	forward	5'- TAGGTACCTTTCAGTCCTTGCCTGCAAACT-3'	*571*
	reverse	5'- TAACGCGTAACAGAGTGTTCTACAGGTTCCA-3'	
rs73746499<br>rs73746499	forward	5'- TAGGTACCTTTCAGTCCTTGCCTGCAAACT-3'	*571*
	reverse	5'- TAACGCGTAACAGAGTGTTCTACAGGTTCCA-3'	
rs73748206	forward	5'- TAGGTACCCTTTCTCAGATTCAGTT-3'	*401*
	reverse	5'- TAACGCGTACCACTGTTTTACAATTGCC-3'	

C) EMSA

SNP			*Probe Sequence*
rs73748206	wild type	F	5'- ATGTTCACATAAGGATGAAATCATCT-3'
		R	5'- AGATGATTTCATCCTTATGTGAACAT-3'
	variant	F	5'- ATGTTCACATAAAGATGAAATCATCT-3'
		R	5'- AGATGATTTCATCTTTATGTGAACAT-3'
