# Supplementary material for: Contribution of FKBP5 Genetic Variation to Gemcitabine Treatment and Survival in Pancreatic Adenocarcinoma
Source: PLoS One. 2013 Aug 1;8(8):e70216. doi: 10.1371/journal.pone.0070216 (PMC3731355; doi:10.1371/journal.pone.0070216)
Supplement: Table S5 — (PDF) [file pone.0070216.s008.pdf]

Table S5. Novel insertions/deletions (Indels) detected by Next Generation resequencing analysis of pancreatic patients DNA samples.

Novel Indels (Insertions/Deletions) Detected by NextGen Analysis in Pancreatic Samples Cohort	
**Location; hg19**	**In/del Type**
35679998	deletion of G
35672457	deletion of AGTA
35663418	insertion of TTT
