# Supplementary material for: Contribution of FKBP5 Genetic Variation to Gemcitabine Treatment and Survival in Pancreatic Adenocarcinoma
Source: PLoS One. 2013 Aug 1;8(8):e70216. doi: 10.1371/journal.pone.0070216 (PMC3731355; doi:10.1371/journal.pone.0070216)
Supplement: Table S6 — (PDF) [file pone.0070216.s009.pdf]

Table S6. List of top window SNPs from sliding window association analysis with avarage FKBP5expression and overall survival. In bold are the SNPs selected for functional characterization. SNP, single nucleotide polymorphism; Chr., chromosmal.

Top Window SNPs from Sliding Window Association Analysis					
**Average Expression**		**Overall Survival**			
#	***SNP Chr. Location***	#	***SNP Chr. Location***	#	***SNP Chr. Location***
1	35780536	1	35721310	26	35701961
2	35780435	2	35721176	27	35701836
3	35779935	3	**35720889**	28	35700795
4	35777852	4	35719458	29	35700518
5	35777525	5	35718531	30	35699311
6	35777278	6	35718318	31	35698369
7	35776478	7	35718242	32	35698253
8	35776462	8	35717519	33	35698135
9	35776273	9	35716324	34	35696209
10	35776119	10	35716217	35	35694697
11	35774523	11	35715938	36	35693257
12	35773746	12	**35715933**	37	35692412
13	35772983	13	35715599	38	35691495
14	35772111	14	35715307	39	35691301
15	35771396	15	35714379	40	35690739
16	35771004	16	35714013	41	35689864
17	35769956	17	35712673	42	35689604
18	35769207	18	35711567	43	35688514
19	35768679	19	35709774	44	35688376
20	35768574	20	35709507	45	35687523
21	35767825	21	35707732	46	**35686829**
22	35766403	22	35705868	47	**35686808**
23	35764231	23	35705681	48	35686162
24	35763962	24	35703459	49	35684834
25	35760898	25	35702644	50	35684479
